# Antihypertensive and Angiotensin-I-Converting Enzyme (ACE)-Inhibitory Peptides from Fish as Potential Cardioprotective Compounds

**DOI:** 10.3390/md17110613

**Published:** 2019-10-29

**Authors:** Soheila Abachi, Laurent Bazinet, Lucie Beaulieu

**Affiliations:** 1 Institute of Nutrition and Functional Foods (INAF), Université Laval, Quebec, QC G1V 0A6, Canada; soheila.abachi-hokmabadi-nazhad.1@ulaval.ca (S.A.); Laurent.Bazinet@fsaa.ulaval.ca (L.B.); 2 Department of Food Science, Faculty of Agricultural and Food Sciences, Université Laval, Quebec, QC G1V 0A6, Canada

**Keywords:** cardiometabolic-syndrome, hypertension, high blood pressure, ACE-inhibitory peptide, antihypertensive, fish, hydrolysate

## Abstract

The term metabolic/cardiometabolic/insulin resistance syndrome could generally be defined as the co-occurrence of several risk factors inclusive of systemic arterial hypertension. Not only that organizations, such as the world health organization (WHO) have identified high blood pressure as one of the main risk factors of the cardiometabolic syndrome, but there is also a link between the occurrence of insulin resistance/impaired glucose tolerance and hypertension that would consequently lead to type-2 diabetes (T2D). Hypertension is medicated by various classes of synthetic drugs; however, severe or mild adverse effects have been repeatedly reported. To avoid and reduce these adverse effects, natural alternatives, such as bioactive peptides derived from different sources have drawn the attention of researchers. Among all types of biologically active peptides inclusive of marine-derived ones, this paper’s focus would solely be on fish and fishery by-processes’ extracted peptides and products. Isolation and fractionation processes of these products alongside their structural, compositional and digestion stability characteristics have likewise been briefly discussed to better address the structure-activity relationship, expanding the reader’s knowledge on research and discovery trend of fish antihypertensive biopeptides. Furthermore, drug-likeness of selected biopeptides was predicted by Lipinski’s rules to differentiate a drug-like biopeptide from nondrug-like one.

## 1. Introduction 

Cardiometabolic syndrome (CMS) is defined as a cluster of several risk factors by various health organizations which marginally varies among all (Table 1) [1]. These risk factors according to WHO manifest in subjects with insulin-resistance of normal glucose tolerance, 10%, subjects of impaired fasting glucose/impaired glucose tolerance, 50%, and subjects of T2D, 80% [2]. These risk factors are undoubtedly interconnected with almost identical underlying mediators, mechanisms and pathways (evident prothrombotic and pro-inflammatory state) that subsequently lead to the development of cardiovascular diseases (CVD) and diabetes [3]. Hence, global mortality rates associated with CVD, as the leading cause of death, were as high as 17 million in 2012 [4]. Along with CVD, diabetes-associated complications as the fourth/fifth causes of death worldwide claimed the lives of 3.2 million in 2003 [4]. 

Adapted from Grundy et al. [1] Subjects diagnosed with metabolic syndrome are two-times as likely to face death, five-times as likely to develop T2D, and three-times as likely to experience a heart attack or stroke in comparison to subjects without the syndrome [3]. Statistics estimate 20–25% of metabolic syndrome cases among the world’s adult population and the same trend is accordingly observed among North Americans [4]. During the past two decades number of T2D cases have nearly doubled due to inappropriate dietary habits (e.g., a diet high in saturated fat and cholesterol) and lifestyle (with 1.1 billion overweight and 312 million obese adults) [5]. In the year 2015, 415 million cases of T2D have been reported by International Diabetes Federation that is predicted to rise to 642 million by 2040 with the estimated cost of 500 billion dollars annually for the health care system, worldwide [6]. These estimates have been confirmed by non-communicable diseases (NCD) risk factor collaboration and WHO. Not only that metabolic syndrome greatly distresses the life expectancy of individuals, but also enforces significant economic burden on societies (Table 2) [7]. According to Lind et al., 25–47% of the subjects with hypertension happen to be diagnosed with insulin resistance/impaired glucose tolerance [8]. High blood pressure alone, as a remediable factor, had been responsible for 6% of the deaths in 1997 worldwide [8,9,10]. Hypertension has been recognized by the National Cholesterol Education Program Adult Treatment Panel III (NCEP-ATP III) and WHO as one of the main risk factors leading to CMS (Table 1) [1].

Hypertension has a broad definition not only outlining blood pressure, but also its association with functional and structural cardiac and vascular abnormalities which consequently damage the target organs (heart, kidneys, brain, vasculature and other organs) causing premature morbidity and mortality [11]. Blood pressure affecting 25–30% of developed countries’ population, as the conventional biomarker, now is announced as an element of a subject’s total cardiovascular risk rather than as a sole risk factor [11,12]. According to 1998 USA statistics, high blood pressure distresses 24–31% of its population enforcing $108 billion on the health care system [13]. Worldwide, in 2000, hypertension affected approximately 972 million and is forecasted to reach 1.56 billion by 2025 [14]. Furthermore, pre-hypertension, an inflammatory associated condition initiating a more severe hypertensive state, is affected by alterations of the immune system [15]. Cytokines, interleukin 6 (IL-6) and tumor necrosis factor alpha (TNF-α), which regulate the production of high-sensitivity C-reactive protein (hsCRP) correspondingly are linked to high blood pressure and obesity [12]. TNF-α and IL-1β levels, counter regulated by IL-6 and soluble TNF-α receptor/soluble TNF-α receptor 2 (sTNFR1/sTNFR2) by TNF-α, correlate with impaired endothelium-dependent dilatation and induction of insulin resistance followed by high blood pressure, respectively [16]. Systemic and chronic mild (low grade) inflammation impairs vasodilation mechanism leading to endothelial dysfunction which is a predecessor factor in the co-occurrence of dyslipidemia, impaired fibrinolysis, and hypertension that are ascribed to insulin resistance and subsequently obesity, as well as diabetes [13,15].

**Figure 1:**
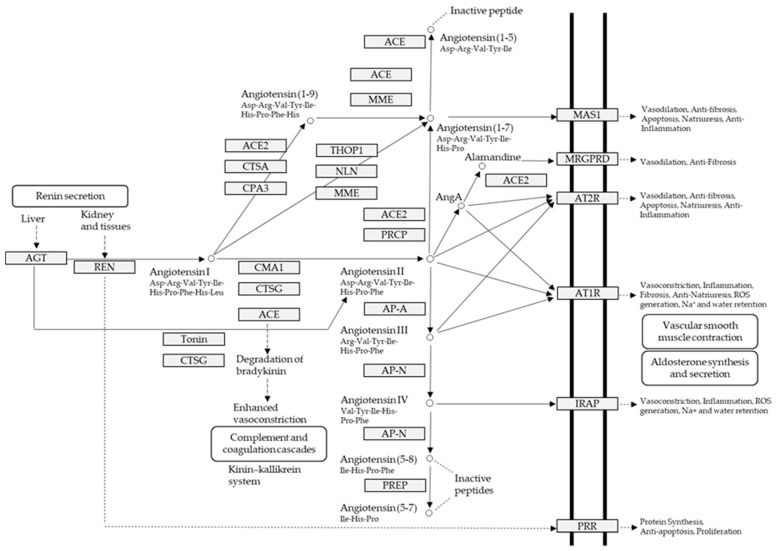
Human renin-angiotensin system pathway map. AGT: Angiotensinogen, ACE2: Angiotensin I converting enzyme 2, AngA: Angiotensin A, AP-A: Glutamyl aminopeptidase, AP-N: Alanyl aminopeptidase (membrane), AT1R: Angiotensin II receptor type 1, AT2R: Angiotensin II receptor type 2, CMA1: Chymase 1, CPA3: Carboxypeptidase A3, CTSA: Cathepsin A, CTSG: Cathepsin G, IRAP: Leucyl and cystinyl aminopeptidase, MAS1: MAS1 proto-oncogene, G protein-coupled receptor, MME: Membrane metalloendopeptidase, MRGPRD: MAS related GPR family member D, NLN: Neurolysin, PRCP: Prolylcarboxypeptidase, PREP: Prolyl endopeptidase, PRR: ATPase H+ transporting accessory protein 2, REN: Renin, THOP1: Thimet oligopeptidase. Figure 1 has been adapted and modified from KEGG Pathway Maps.

In addition to obesity and diabetes, hypertension is potentially a variable known risk factor for atrial fibrillation which is the major component of stroke and associated mortality; nevertheless, its pathophysiological mechanism is not yet well understood [12]. Inflammation, on the one hand, is a catalyst for the structural changes of the left ventricle and the left atrium caused by atrial fibrillation; on the other hand, it is important in inducing the production of angiotensin II, as well as enhanced activity of renin-angiotensin-aldosterone system (RAAS) (Figure 1) [12,17]. Blood pressure, high blood pressure and left ventricle hypertrophy incidences are directly related to high amounts of hsCRP which are produced in chronically inflamed tissues [12]. Inflammatory infiltrates, and oxidative damage upregulate the expression of angiotensin-II receptors causing increased atrial cell death and leukocyte infiltration that are clearly found within the arterial tissue in atrial fibrillation cases [12,18]. Albeit, there is more evidence on the association of atrial fibrillation with heart’s inflammatory and systemic inflammatory diseases (myocarditis, pericarditis, psoriasis). 

Several classes of drugs; direct inhibitors of the renin-angiotensin system (angiotensin-converting enzyme inhibitors, angiotensin-receptor blockers), adrenergic beta-antagonists, diuretics, sodium chloride symport inhibitors, and calcium channel blockers, are prescribed for the treatment of hypertension, though, accompanying side-effects limit their practice in medicine. For instance, upon ingestion of angiotensin-I-converting enzyme (ACE) inhibitors patient may encounter severe or mild adverse effects, such as cough, headache, diarrhea, dizziness, fatigue, angioedema, hyperkalemia or, in rare cases, renal and cardiac failure [19]. Different classes of drugs are suggested, corresponding to the indication of hypertension. For example, with an indication of heath failure diuretics, beta-blockers, ACE inhibitors, angiotensin receptor blockers, calcium channel blockers and aldosterone antagonists are the first-line medications recommended for the effective management of hypertension [20]. For chronic kidney disease indication, ACE inhibitors, angiotensin receptor blockers, and calcium channel blockers are suggested to bring the blood pressure under control [20]. New pharmaceutical ingredients are designed in a way to target the inflammatory processes and modulations of its pathways (RAAS-blockers and statins) for the treatment of hypertension and related disorders [21]. In agreement cholesterol level is not any longer recognized as dyslipidemia treatment goal for reduction of atherosclerotic cardiovascular risk in 2013 published guidelines of the American Heart Association and American College of Cardiology. Correspondingly, CVD risk prediction algorithms are now based on hypertension, and not on low-density lipoprotein anymore, increasing the sum of combination therapy (e.g., RAAS-blockers and statins) prescribed patients [22]. 

**Figure 2:**
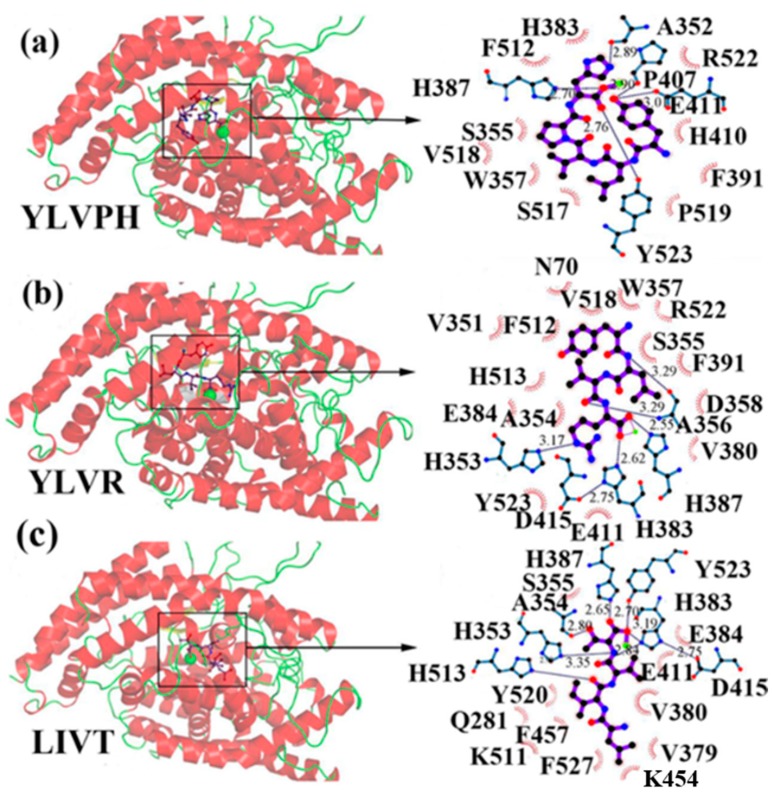
3D plots of the frontier molecular orbital of three food-extracted anti-ACE (angiotensin-I-converting enzyme) biopeptides. Per docking results Tyr-Leu-Val-Pro-His occupied an active groove surrounded by Ala352, Glu411, Tyr523, and His387 (His387, His512, Ser355, Val518, Trp357, Ser517, Pro519, Pro391, His410, and Arg522 having van der Waals contacts with the penta-peptide YLAPH), Tyr-Leu-Val-Arg occupied an active groove surrounded by His353, Ala356, His383, and His387 (Asn70, Val518, Trp357, Arg522, Val351, Phe512, and Ser355 having van der Waals contacts with the tetra-peptide, YLVR), Leu-Ile-Val-Thr occupied an active groove surrounded by Ala356, His353, His383, His387, His513, Tyr523 (Ser355, Glu411, Tyr520, Gln281, Phe457, Lys511, Phe527, Lys454, Val379, and Val380 having van der Waals contacts with the tetra-peptide, LIVT). Adapted and modified from Fang et al. [32].

In the concept of prevention and treatment of CMS associated risk factors inclusive of hypertension, natural products, such as peptides have shown promising effects. Many studies have shown that bioactive peptides from animals (terrestrial, marine), and plant sources, in addition to their many health effects, could also be utilized in the effective management of hypertension, a key factor of cardiometabolic diseases occurrences [23,24,25,26,27,28,29]. ACE inhibition activity of food-derived peptides, inclusive of fish-extracted ones, are primarily by competitive inhibition mode rather than non- and or uncompetitive modes [28,30,31]. Food-originated anti-ACE biopeptides bind to the enzyme and hinder its activity, bringing the blood pressure under control. For example, in a study by Fang et al. interactions of three food-isolated oligopeptides with ACE have thoroughly been studied, by computational modeling, elucidating different inhibition mechanisms of antihypertensive biopeptides (Figure 2) [32]. The majority of anti-ACE peptides are not specific to one family of an animal or a plant and can certainly be found in many other species, e.g., antihypertensive salmon di- and tri-peptides with comparable animal-, plant-, marine- and or insect-origin sequences (Table 3). For instance, fractions and subfractions of jellyfish enzymatic hydrolysate have hindered the ACE activity at IC_50_ as low as 1.28 mg mL^−1^ and 0.16 mg mL^−1^ respectively in an in-vitro experimental model [33]. The most potent penta-peptides of the same hydrolysate, isolated by gel and high-performance liquid chromatography (HPLC) with polar amino acids at the C-terminal, also had blood pressure (BP) decreasing effect in spontaneously hypertensive rats (SHR) when administered orally at the concentration of 10 mg kg^−1^ body weight (BW) [33]. The above is only an example of many marine animals including many fish types with anti-hypertension activities [34]. Varying fish-derived biopeptides, in terms of MW, length and composition, have inhibited the activity of ACE and controlled blood pressure successfully in the in-vitro and or in the in-vivo experimental settings, as well as human studies. Anti-ACE fish biopeptides, similar to other bioactives, mostly exert their effect on the subject non-cytotoxically, negligible and or zero cytotoxicity, making them superior to the synthetic drugs for treatment and prevention of hypertension. These biopeptides can be produced by digestion, isolation, and purification processes, affecting the structural, compositional and characteristics of the final product, discussed further in the following sections. Authors herein have solitary tried to bring together more than twenty studies directed on fish-extracted peptides or fish-originated products demonstrating their antihypertensive and or ACE inhibitory effects in different experimental settings and models.

## 2. Biological Activity of Fish, Fish By-Product Protein and Peptides on Hypertension 

**Figure 3:**
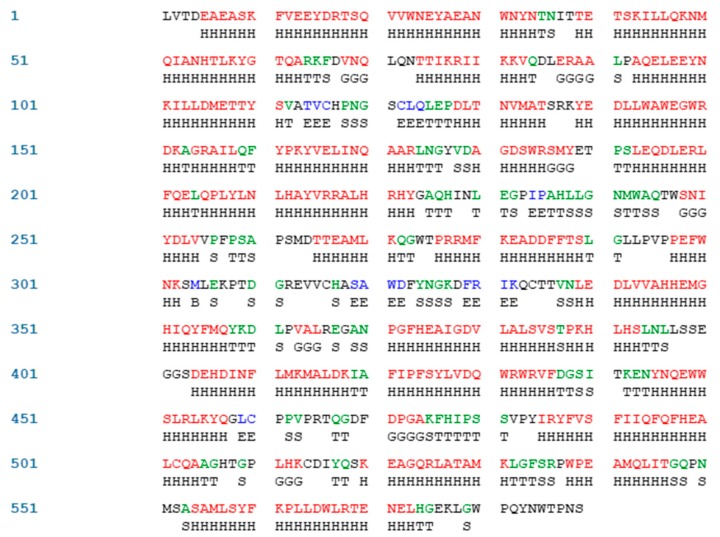
Sequence and secondary structure of chain A human ACE (PDB code: 1O8A). Active sites, according to Attique et al. are; His317, Ala318, Ser319, His347, Glu348, His351, Glu375, Phe421, Lys475, Phe476, His477, Val482, Tyr484, Tyr487.

ACE (with alternative names of dipeptidyl carboxypeptidase I and kininase II), a carboxydipeptidase, deactivates bradykinin (a vasodilator) and controls blood pressure through converting angiotensin-I (an inactive decapeptide prohormone, Asp-Arg-Val-Tyr-Ile-His-Pro-Phe-His-Leu) to angiotensin-II (a potent octapeptide vasoconstrictor, Asp-Arg-Val-Tyr-Ile-His-Pro-Phe) stimulating the release of aldosterone, and thus, increasing blood pressure (Figure 1) [13]. Physiochemical properties of human ACE (predicted by ProtParam), its active sites and sequence are listed, and shown in Table 4 and Figure 3 [37]. ACE inhibitors, e.g., captopril, are regularly used to treat hypertension, and other cardio-related diseases as an influencer of blood pressure [21]. Proline moiety of captopril interacts with ACE active site, primarily through two histidine residues, inhibiting its activity [38]. Binding sites of captopril on ACE have been observed at Trp67, Asn68, Thr71, Asn72, Met340 and Arg348 residues [39]. Generally, the proline residue, in most of the captopril alike anti-ACE drugs, e.g., enalaprilat, zofenoprilat, fosinoprilat, etc., impedes the enzyme’s activity [38].

Synthetic ACE inhibitors impose rather significant side effects, such as a dry cough and/or contradiction with other medications on the subjects, therefore, natural plant, animal, and marine-based alternatives have attracted great attention recently [35,40]. Sulfhydryl-containing, dicarboxylate-containing, and phosphonate-containing agents are the main groups of ACE inhibitors [41]. Among many types of bioactive compounds, peptides from various sources, such as bovine, egg, milk and plants have been the subject of many studies, and shown biological activity against hypertension, e.g., di- and tri-peptides of different sources with matching salmon-extracted sequences (Table 3) [35]. Kumar et al. have extensively reviewed natural ACE inhibitors [35]. Among marine-based peptides whether plants or animals, fish bioactive peptides derived using various extraction, isolation and fractionation techniques have also been investigated for their anti-hypertensive activities, such as ACE inhibition (Table 5 and Table 6). Of all the farmed and wild fish species, many carp, salmon, tilapia, sardine, and tuna have been analyzed for their biological activities (Table 6). These species’ proteinaceous portion contains not only the essential amino acids possessing all the parameters of a good food, but also their associated hydrolysates have countless health benefits inclusive of antihypertension effects (Table 6). 

In an in-vivo experimental setting, after two hours (h) treatment, tested grass carp material showed significant antihypertensive activity (43 mmHg systolic blood pressure (SBP) maximal drop at the dosage of 100 mg kg^−1^ body weight (BW)) in SHR compared to the control group [42]. Grass carp peptides of size 725–1228 Da, rich in leucine, aspartic acid, phenylalanine and glycine amino acids, at 100 mg kg^−1^ BW in SHR 6 h post-administration significantly reduced SBP of approximately 30 mm Hg, in comparison to the control group (captopril), the medication of choice, with similar effect at dosage of 10 mg kg^−1^ BW) and the effect persisted for several weeks after the initial administration [43]. In an in-vitro study, hydrolysates of salmon pectoral fin by-products (digested by alcalase, flavourzyme, neutrase, pepsin, protamex and trypsin) that were isolated and fractionated to various peptide fractions by consecutive chromatography, demonstrated competitive, non-competitive and mixed inhibition modes against ACE in a dose-dependent manner with IC_50_ values of 7.72–10.77 μM [44]. For the extraction and hydrolysis of intended peptides, selection of enzymes and the subsequent fractionation and purification techniques are vital considerations, since these factors govern the structure of the final product and its mode of action in the target site (Table 5).

## 3. Impact of Enzymatic Digestion and Purification Processes on Antihypertension and ACE Inhibition Activity 

The review of Lee et al., focusing on animal-, marine-, and plant-origin ACE inhibitors, have concluded that optimal hydrolysis treatment depends on the type of the protein source [23]. Optimal hydrolysis treatment for different food sources have been postulated as; animal-extracted enzymes (e.g., pepsin, trypsin, chymotrypsin) for animal products, and microbe-extracted enzymes (e.g., alcalase, neutrase, thermolysin) for marine organisms and plants [23]. In addition to enzymatic hydrolysis, solvent extraction has also efficaciously been unitized for production of plant-based ACE-inhibitors [24]. The proteolytic enzymes from different sources (from animals’ digestive tract, microbes and other natural sources), including autolysis in which naturally occurring enzymes are actively engaged in the fermentation process, have overall been used for the extraction of ACE-inhibiting peptides from marine-organisms [34]. In accordance, of the above-mentioned digestion processes, enzymatic hydrolysis has commonly been used in the preparation of fish-origin antihypertensive and anti-ACE hydrolysates, with the exception using water-extraction procedure which has shown to be an effective method for isolation of mushroom-based ACE-inhibitors (Table 5) [24,49]. 

Following digestion, in general, hydrolysates are further processed to obtain a purer, and likely, a more potent product. Next, products are subjected to filtration. This commonly is practiced for every type of food source. Subsequently, a chromatographic technique, or combination of few, would be used for separation of the peptides based on their many structural and compositional characteristics (Table 5). A clear example is that ACE-inhibition activity of jellyfish hydrolysate was enhanced by 8-fold (from IC_50_ of 1.28 mg mL^−1^ to the low end of 0.16 mg mL^−1^) by desalting and gel column chromatographing [33]. Ultra-filtering of grass carp lysate with 10 kDa molecular weight MWCO ultrafiltration (UF) membrane improved IC_50_ by about 3-fold (from 0.692 mg mL^−1^ to 0.272 mg mL^−1^) and further desalting by about 7-fold (from 0.692 mg mL^−1^ to 0.105 mg mL^−1^) [42]. Even so, precipitating and desalting, frequently applied to plant- and marine-based hydrolysates, is not so common for fish-hydrolysates [24,33,42]. Separation and purification of these molecules are well necessary for the characterization and identification of the biopeptides, in terms of amino acid sequences and molecular mass (Table 5). 

### 3.1. Enzymatic Digestion 

Depending on the amino acid composition and the peptide bonds of the selected sample, food source, many different enzymes could be used to produce the most preferred potent bioactive molecule(s). An enzymatic reaction, depending on the type of enzyme and the substrate, results in final products with diverse functionalities (Table 5 and Table 6).

#### 3.1.1. Effect of Enzymes on Different Peptide Bonds 

Alcalase and neutrase, both, produce peptides with hydrophobic amino acids at the C-terminus. Alcalase, an esterase, explicitly produces short peptides (usually < 10 kDa) with hydrophobic amino acids at the C-terminal, as well as heterocyclic amino esters, whereas, neutrase, neutral zinc metallo endo-protease, is mainly specific to leucine, tyrosine, tryptophan and phenylalanine [54,78,79,80]. On the other hand, papain with broad specificity cleaves and catalyzes peptide bonds of basic amino acids, leucine, methionine, tryptophan, glycine, tyrosine, phenylalanine and amino acid ethyl ester residues with a preference for hydrophobic amino acid large side-chains contrary to alcalase, which is evident in numerous studies [33,54,78,79,80,81]. The chymotrypsin, like papain, cleaves bulky side chains, rather non-polar amino acids, with valine, alanine, leucine, proline, tyrosine, phenylalanine, histidine, and tryptophan at C-terminal [57,60,63]. Flavourzyme, an exopeptidase with exocatalytic action, generates a great deal of amino acids, as well as long chain peptides with higher MW (>19 kDa in the hydrolysis of poultry meals and flaxseed) [82,83]. Proteinase K, a serine protease, cuts at the peptide bond site next to the carboxylic group of aliphatic, aromatic amino acids with a preference for hydrophobic amino acids comparable to alcalase and neutrase [54,84].

Due to improved enzymatic endo-actions and specific hydrolysis of carboxyl-terminal hydrophobic amino acids, alcalase has been preferred over other common commercial and non-commercial enzymes for the extraction of ACE inhibitory peptides specifically from grass carp and other fish types [42,44,45,85]. According to Lee et al. alcalase may not be suitable for the digestion of every fish type [57]. For the extraction of ACE inhibitors from dried bonito, over a broad range of enzymes, thermolysin was the most superior [60]. Consecutive digestion of the peptic hydrolysate of dried bonito by digestive proteases affected the effectivity of peptides, nonetheless, the same method had a non-significant effect on the additional digestion of thermolysin hydrolysate [60]. The trypsin/chymotrypsin and chymotrypsin increased the potency of the peptic hydrolysate by 19% (IC_50_ 38 µg mL^−1^) and 13% (IC_50_ 41 µg mL^−1^), respectively, nevertheless trypsin alone decreased this effect by 38% (IC_50_ 65 µg mL^−1^) in comparison to the pepsin hydrolysate (IC_50_ 47 µg mL^−1^) [60]. 

#### 3.1.2. Effect of Enzymes on the Separation of Blood Pressure Lowering Peptides

In the study of Ghassem et al., the biological activity of the enzymatic hydrolysates, from snakehead fish, in decreasing order were; alcalase > proteinase k > flavourzyme > neutrase > papain in comparison to the crude sarcoplasmic snakehead fish muscle protein with the least and alcalase hydrolysate with highest activities [54]. A similar approach was followed by Wijesekara et al. where muscle protein of seaweed pipefish was digested through enzymatic hydrolysis by various commercial enzymes, and all the resultant hydrolysates exhibited anti-ACE effect, ~70–86%, in decreasing order of alcalase > trypsin > papain, pepsin > neutrase > pronase [55]. Bonito hydrolysates all affected the ACE activity to some degree, IC_50_ ranging from 29 µg mL^−1^ to 175 µg mL^−1^, in decreasing order of thermolysin > trypsin/chymotrypsin digested peptic hydrolysate > chymotrypsin digested peptic hydrolysate > pepsin > trypsin digested peptic hydrolysate > chymotrypsin > trypsin > trypsin/chymotrypsin [60]. Tuna fish frame proteins, when processed by specific and non-specific commercial enzymes, demonstrated ACE inhibition activity to varying degrees, ~45–88%, and the effects in decreasing order of activity were; pepsin > α-chymotrypsin > neutrase > alcalase > trypsin > papain [57]. 

### 3.2. Isolation and Purification

Moreover, of the above-discussed procedures and techniques, mainly membrane filtration and chromatographic techniques are in use for isolation and purification of anti-hypertensive and anti-ACE biopeptides from fish lysates (Table 5). While smaller MWCO ranges are applied for plant-based hydrolysates (1–10 kDa), a wider range of 1 kDa up to 30 kDa has been adapted for fish hydrolysates [24]. Comparable chromatographic techniques (e.g., size exclusion chromatography (SEC), ion (cat- and an-ion) exchange chromatography (IEC), solid phase extraction (SPE), reversed phase high performance liquid chromatography (RP-HPLC) are practiced for fish- and plant-based hydrolysates (Table 5) [24]. Derived products significantly vary from one another in terms of MW, chain length, structure and composition, which would be influential on the properties of the peptide and subsequently its bioactivity (Table 5, Table 6 and Table 7). 

#### Effect of Different Fractionation Processes on the Separation of Blood Pressure Lowering Peptides

In a study by Chen et al. fresh grass carp was primarily hydrolyzed by two commonly used enzymes, alcalase and neutrase. Subsequently, peptides were isolated by UF process, MWCO 10 kDa, and the resultant fraction was further desalted using mixed ion exchange resins [42]. Desalted permeate had significantly higher ACE inhibition activity (~2–6 times higher) than the permeate of UF, the initial hydrolysate and the reference protein, soya peptide [42]. Molecular weight distribution analyses of the desalted UF permeate fraction revealed that > 90% of the peptides were of sizes 190–1000 Da [42]. Amino acid composition analysis results showed that glutamic- and aspartic-acid (negatively charged hydrophobic amino acids) are the most dominant amino acids in the fraction and the abundance of amino acids follow similar trends in both the hydrolysate and the desalted protein [42]. 

In accordance, fish scale peptides of the same species that were hydrolyzed using neutral protease and isolated by macroporous resins resulted in hydrophobic ACE inhibitory di- to penta-peptides of sizes 145 Da and 650 Da [46]. Another ACE inhibitory tri-peptide from whole fresh grass carp with MW of < 3 kDa was hydrolyzed by alcalase, afterwards isolated and purified sequentially using UF, macroporous adsorption resin, and two steps of RP-HPLC [45]. Alcalase and protamex digested salmon hydrolysate at 1 mg mL^−1^ inhibited 56% and 50% of the ACE activity, respectively, whereas, others’ effect was below 23% [44]. Molecular mass distribution of salmon trimming hydrolysate showed that higher percentage of corolase-derived peptides, about 44%, were < 5 kDa in comparison to different enzyme preparations, and evidence has confirmed the improved bioactivity of smaller size peptides [50]. In general, alcalase and corolase release more of short proline-rich hydrophobic amino acids, also observed in the study of Neves et al., are well known for their anti-ACE attributes [50,86,87]. In agreement, salmon alcalase-digested hydrolysate was about 97% rich in peptides with MW of <10 kDa [50,86,87].

The fractionation of tilapia by chromatographic techniques, four-step purification, significantly affected the ACE inhibition activity of the different fractions obtained throughout the process [53]. The most potent ACE inhibitory peptide of the tilapia, IC_50_ of 0.15 mg mL^−1^, was obtained by SEC in a successive fractionation process [53]. Successive purification of tuna frame protein peptic hydrolysate by chromatographic techniques resulted in a semi-pure product (PHII-F3-3) which was ~20-times more potent in comparison to the crude hydrolysate [57]. One of the three fractions (F3) from alcalase protein hydrolysate of the seaweed pipefish using FPLC on DEAE FF anion exchange column was significantly active with IC_50_ of 0.068 mg mL^−1^ [55]. F3 was sub-fractionated by RP-HPLC on C_18_ column to three fractions with the IC_50_ values of 0.62 mg mL^−1^ and 1.44 mg mL^−1^ for F3-II and F3-III, respectively [55]. Evidently, the peptides in the hydrolysate have a synergistic effect as F3 is 9-times and 21-times more potent than its sub-fractions, the F3-II and F3-III, correspondingly [55]. Ultrafiltration, MWCO 5 kDa, non-significantly improved anti-ACE effect by 3% only (88% ACE inhibition with 0.14 mg peptide concentration) in comparison to the 85% inhibition of crude hydrolysate of tilapia (*Oreochromis niloticus*) by 0.51 mg of peptides, followed by DEAE-Sephacel anion exchange chromatography fraction (59% at 0.07 mg of peptides), CM-Sepharose cation exchange chromatography fraction (38% at 0.02 mg of peptides), and Superdex peptide 10/300 GL size exclusion chromatography fractions with least effectivity (35% at 0.01 mg of peptides) [53]. Use of diverse lysing, separating and purifying procedures lead to different cleavage sites, length and amino acid types of a peptide chain, consequently affecting the bioactivity of the final product (Table 5 and Table 6).

## 4. Structure-Activity Relationship 

Scientists are in general agreement of a strong association between the ACE-inhibition and or anti-hypertensive effect of a biopeptide with its structure; size, chain length, type and order of amino acids in the sequence. Conclusively decades ago, Cheung et al. declared the existence of a hydrophobic amino acid at either of the peptides’ terminals vital for ACE-inhibition activity of a peptide, namely, phenylalanine, proline at C-terminal and isoleucine, valine at N-terminal of the biopeptide [88]. In agreement with Cheung et al., phenylalanine exists in a great deal of fish blood pressure controlling peptides, either at C- or N- terminal (18 sequences in total), of varying masses and lengths (Table 6 and Table 7). Even so surprisingly years later, Daskaya-Dikmen reviewing plant-derived ACE-inhibitors, though agreeing on the importance and relationship between ACE-inhibition activity and peptide structure, could not specifically conclude on the structural and compositional feature of a potent blood pressure lowering biopeptide [24].

Cushman et al. back in 1971, suggested the possible interaction of ACE active sites with tyrosine, tryptophan, proline, phenylalanine and one hydrophobic amino acid at the C-terminal residue to be responsible for the ACE inhibition activity of the potent peptides [87]. In agreement with the proposition of Cushman et al. amino acid sequences of the potent fractions of salmon by-product hydrolysate with hydrophobic amino acids at the C-terminal residue were Val-Trp-Asp-Pro-Pro-Lys-Phe-Asp, Phe-Glu-Asp-Tyr-Val-Pro-Leu-Ser-Cys-Phe, and Phe-Asn-Val-Pro-Leu-Tyr-Glu [44,87]. In general, salmon muscle dipeptides with phenylalanine in their structure exhibited the significant activity of which Ile-Phe, at 50 µM with 86% inhibition was the most compelling [51]. The dipeptide Ile-Phe has repeatedly been reported as an anti-hypertensive peptide of animal and plant source (e.g., chicken, pork, bovine, egg, soybean, etc.) (Table 3). Presence of same Ile-Phe in animal-, plant- and marine-origin oligopeptide sequences also have been shown, per AHTPDB database, to effectively prevent hypertension (available at http://crdd.osdd.net/raghava/ahtpdb/index.php). The dipeptides Val-Phe and Leu-Phe were the most abundant active peptides in the salmon muscle hydrolysate constituting 0.12% and 0.06% of the total content, respectively [51]. Comparable peptides have been frequently identified in sources other than fish with potent blood pressure lowing effects (Table 3). 

The two isolated and identified penta- and hexa-peptides sequence of snakehead muscle sarcoplasmic protein alcalase hydrolysate, Leu-Tyr-Pro-Pro-Pro and Tyr-Ser-Met-Tyr-Pro-Pro, contained proline and one hydrophobic amino acid at the C-terminal which has earlier been reported to interact with ACE active sites hindering its activity [54,87]. Interaction of skate originated oligopeptides (Met-Val-Gly-Ser-Ala-Pro-Gly-Val-Leu and Leu-Gly-Pro-Leu-Gly-His-Gln) with ACE has been observed at Trp67, Asn68, Thr71, Asn72, Thr74, Glu76, Thr77, Lys338, Asp346 and Arg348 [67]. Looking closely at the pattern of snakehead-extracted anti-ACE pentapeptide (Leu-Tyr-Pro-Pro-Pro; IC_50_ 1.3 μM) and cuttlefish-extracted anti-ACE pentapeptide (Val-Glu-Leu-Tyr-Pro; IC_50_ 5.22 μM) presence of Leu-Tyr-Pro order of amino acids in both sequences is conspicuous (Table 7) [54,66]. Interestingly sequencing of the selected salmon trimmings corolase hydrolysate fractions revealed Tyr-Pro as the most potent ACE inhibitory dipeptide (IC_50_ 5.21 μM) which is in accordance with the scheme of Cushman et al. [50,87]. A further up-close look at snakehead- and cuttlefish-extracted pentapeptides one could observe the occurrence of Tyr-Pro, the most active salmon-extracted anti-ACE dipeptide, in both of the anti-ACE biopeptides [50,54,66]. The sequences of sea broom hydrolysate based on IC_50_ values, 0.003–0.16 mg mL^−1^, in decreasing order were Val-Ile-Tyr > Val-Tyr > Gly-Tyr > Gly-Phe [56]. 

Presence of proline in the sequence of fish-extracted biopeptides may play more vital than MW, chain length and hydrophobicity. Cuttlefish-origin di-peptide, Gly-Ser (163.0 Da, hydrophobicity of +9.51 Kcal mol^−1^), was respectively ~ 220- and 60-times less potent than cuttlefish-origin Val-Glu-Leu-Tyr-Pro penta-peptide and Ser-Thr-His-Gly-Val-Trp hexa-peptide with comparable hydrophobicities (+9.25 and +9.54 Kcal mol^−1^) (Table 7) [66]. In accordance with proline richness and its number of occurrences in a sequence could greatly increase the potency of a biomolecule. Snakehead Leu-Tyr-Pro-Pro-Pro penta-peptide (IC_50_ 1.3 μM), containing three prolines, was ~2-times more active than Tyr-Ser-Met-Tyr-Pro-Pro hexa-peptide (IC_50_ 2.8 μM), containing two prolines, with similar properties (pI, charge and hydrophobicity) (Table 7). It shall be emphasized that scientists agree on the importance of proline moiety in the activity of synthetic ACE inhibitors [38]. However, comparing the two deca-peptides with relatively comparable properties, the occurrence of two phenylalanine (Phe-Glu-Asp-Tyr-Val-Pro-Leu-Ser-Cys-Phe), one at C- and another at N-terminus, appears to exert more effective anti-ACE effect (~ 3-times) than two successive prolines at C-terminus (Leu-Leu-Met-Leu-Asp-Asn-Asp-Leu-Pro-Pro) of a bioactive sequence (Table 7) [44,69].

Existence of three sequential proline residues in a sequence results in a polyproline structure of which the left-handed-polyproline-II helices are more common than their right-handed counterparts. Proline-rich polyproline peptides, especially left-handed structures, have been documented as indispensable for the presentation of antimicrobial, immunomodulation, antioxidant and other bioactivities [89]. Tri-proline peptides engage in protein-protein signaling interactions thus playing important in various cell signal transduction pathways [90]. Vitali et al. have reviewed proline-rich peptides as potential leads for research and development of pharmaceutical compounds [89]. Leu-Tyr-Pro-Pro-Pro may, in that sense, owe its potency to the polyproline structure. Factually, structure-wise, proline and hydroxyproline amino acids, are derivatives of pyrrolidine. Pyrrolidine moiety is common in drug discovery, and its ring structure can be found in many. More than a dozen FDA approved drugs are merely substituted pyrrolidine compounds [91,92]. Interestingly of all the fish anti-ACE and antihypertensive sequences, reviewed herein, there are sixteen peptides with proline at their C-terminal (none at N-terminal) and of those four of the sequences contain 2-3 successive prolines [50,54,69]. Of all, Leu-Tyr-Pro-Pro-Pro (1.3 μM, 585 Da) and Asp-Tyr-Gly-Leu-Tyr-Pro (62 mΜ, 349 Da) were the most and the least potent sequences, based on reported IC_50_ values, with more or less similar properties except MW and number of proline residues (Table 6 and Table 7) [54,60].

Presence of tyrosine (a polar amino acid) and phenylalanine at the C-terminus along with hydrophobic amino acids (valine and glycine) at the N-terminus may contribute to the activity of sea broom-origin di- and tri-peptides [56,87]. The most effective peptide, Val-Ile-Tyr, was 190-times more potent in comparison to the crude sea broom hydrolysate [56]. Existence of tyrosine among fish-isolated antihypertensive fractions is rather common, and fifteen of the isolated peptides happen to contain this amino acid at either of their extremities (mainly at C- and equally at N-terminal or penultimate C-terminal position). Among those fifteen peptides, Tyr-Ser-Met-Tyr-Pro-Pro (IC_50_ 2.8 μM, 756 Da) and Gly-Tyr (IC_50_ 265 μM, 238 Da) were the most and the least active ones [54,56]. 

Dipeptides containing tryptophan at the C-terminal are relatively more potent than their reversed sequence counterparts with tryptophan at the N-terminal [30,51]. Consistently dipeptides (Ile-Trp, Leu-Trp) with tryptophan at C-terminal could withstand the sequential digestive enzymes reaction (pepsin, trypsin, chymotrypsin) of 70% and 61%, respectively, in comparison to the crude salmon peptide with the stability of only 47% [51]. Dipeptides Val-Trp, Ile-Trp, Gly-Trp, Met-Trp, Ile-Trp, Leu-Trp (tryptophan at C-terminal position), Trp-Ala, Trp-Met (tryptophan at the N-terminal position), Phe-Leu, Leu-Phe, Val-Leu, Ile-Leu, Leu-Ile, Val-Phe, Ile-Phe, Tyr-Phe (hydrophobic amino acid at C-terminal position) Phe-Tyr (tyrosine at C-terminal position) along with tripeptides Val-Ile-Phe, Ile-Val-Phe, Phe-Val-Leu, Ala-Phe-Leu, Leu-Val-Leu, Val-Ile-Leu, Ile-Val-Leu, Phe-Ile-Ala, Tyr-Leu-Val, and Ile-Val-Trp (hydrophobic amino acid at C-terminal position) extracted and isolated from salmon and its by-products have been found to be active ACE inhibitors [51]. Most effective salmon muscle peptides were Ile-Val-Trp and Ile-Trp which exhibited 35% and 49% ACE inhibition, at a concentration of 1.0 mM and 1.0 μM respectively in comparison to the crude hydrolysate with IC_50_ of 79 µg mL^−1^ [51].

Two of the tested synthetic tilapia-origin heptapeptides, Met-Ile-Leu-Leu-Leu-Phe-Arg and Leu-Asn-Leu-Gln-Asp-Phe-Arg contained Phe-Arg, which is a sequence previously reported for its prominent ACE inhibitory effect by BIOPEP database (available at http://www.uwm.edu.pl/biochemia/index.php/en/biopep) [53]. Tilapia-extracted hepta-peptides with C-terminal Phe-Arg clusters and parallel MW were effective at relatively low doses; however, Met-Ile-Leu-Leu-Leu-Phe-Arg with a net charge of +1 was ~ 7-times more potent than Leu-Asn-Leu-Gln-Asp-Phe-Arg with a net charge of 0. In addition, their hydrophobicity significantly differed from one another. Met-Ile-Leu-Leu-Leu-Phe-Arg was less hydrophobic (+2.46 Kcal mol^−1^) than Leu-Asn-Leu-Gln-Asp-Phe-Arg (+10.76 Kcal mol^−1^) (Table 7). Looking through AHTPDB database, regardless of peptide extraction source, the occurrence of Phe-Arg cluster is more common at N-terminal of bioactive sequences than C-terminal and or penultimate C-terminal position.

According to Cheung et al., the presence of hydrophobic amino acids, methionine and leucine, at their N-terminal may have correspondingly affected their anti-ACE activity [53,88]. Commonly, positively charged amino acids could contribute to the prevention of ACE activity, hence, l-arginine alone or in combination with lisinopril, an ACE inhibiting drug, has shown antihypertensive effects in addition to its anti-inflammatory attributes [93,94,95,96]. In accordance, no activity was detected when arginine was cut off from the active ACE inhibitor sequence of Met-Ile-Leu-Leu-Leu-Phe-Arg [53]. Slashing off arginine from synthesized peptide sequence (Leu-Asn-Leu-Gln-Asp-Phe-Arg; IC_50_ 0.85 µM, Leu-Asn-Leu-Gln-Asp-Phe; IC_50_ 0.51 µM) improves the anti-ACE activity by 67% as phenylalanine, a hydrophobic amino acid, is then situated at the C-terminal position, which has been repeatedly reported as one of the criteria for an ACE inhibitor peptide [44,53,87]. Arginine was positioned at C-terminal of 70% of the ten identified amino acid sequences of a selected tilapia ACE inhibitory fraction (resulted from SEC in a succeeding fractionation method with masses ranging from 791 Da to 1526 Da) with in-vitro thermo-, pH- and digestive proteases- stability [53]. Arginine is a positively charged amino acid, however, its role in stability, when situated at C-terminus, to digestive enzymes have not thoroughly been elucidated. Quirós et al. modified a hexapeptide of mammal-origin β-casein, Leu-His-Leu-Pro-Leu-Pro, for better understanding of the structure activity relationship and found out that substitution of proline at C-terminus by arginine and leucine in the penultimate position by glycine not only improved its antihypertensive activity by double, but also enhanced its stability to gastrointestinal enzymes, respectively [97]. To the author’s knowledge, a comparable study on a fish-origin peptide has not yet been directed. In this review, there are eight C-terminal and one N-terminal arginine fish-derived peptides (Table 6 and Table 7).

The MW of the two hepta-peptides isolated and identified from tilapia hydrolysate are insignificantly different from one another, however, Met-Ile-Leu-Leu-Leu-Phe-Arg (905.83 Da) with Leu-Phe, Leu-Leu-Phe and Phe-Arg ACE inhibitory peptide sequences in its chain was 7-times more effective than the other (Leu-Asn-Leu-Gln-Asp-Phe-Arg; 905.66 Da) indicative of the fact that a combination of factors (size, sequence and digestive stability) are required for the bioactivity and potency of a peptide [53]. Another pentapeptide, Leu-Lys-Pro-Asn-Met, from dried bonito with proline situated inside the sequence could only minimally resist the same digestion, 11%, while a sardine dipeptide which has also been isolated from scales of sea bream, Val-Tyr, without proline in its sequence exhibited higher stability, 32%, due to its small size [51,56]. ACE inhibition, however, may be of a synergetic type effect as with tilapia hydrolysate derived fractions that of all only few of the synthesized peptide sequences hindered the enzyme’s activity [53]. 

MW is not always indicative of biological activity, since purified PHII-F3-3 is a relatively heavy peptide, 21- amino acids chain and MW > 2 kDa, thus, has shown perceptible in-vitro and in-vivo anti-hypertensive effects against high blood pressure, non-competitively binding to non-active sites of ACE [57]. One of the final four resultant fractions of SEC, the final stage of successive fractionation, that was the most biologically active ACE inhibitory peptide with IC_50_ of 0.15 mg mL^−1^ was subjected to amino acid sequence analysis [53]. The MW of the sequences varied from 677 Da up to 1526 Da [53]. Lowest MW did not necessarily denote the highest potency of the peptide, since most potent sequence was Met-Ile-Leu-Leu-Leu-Phe-Arg with MW of 905 Da and IC_50_ of 0.12 µM [53]. Contrary to the study of Toopcham et al., MW of the identified hexapeptides, from alcalase hydrolysate of the seaweed pipefish, was characteristic of their biological activity, thus, the smallest peptide, F3-II with MW of 744 Da, was non-cytotoxically more potent than F3-III with MW of 917 Da [53,55]. Identified hexapeptides of alcalase peptidic hydrolysate of the seaweed pipefish have MW of < 1000 Da with hydrophobic (Thr-Phe-Pro-His-Gly-Pro) and positively charged (His-Trp-Thr-Thr-Gln-Arg) amino acids at C-terminal of the sequence which is in line with previous studies [53,55,93,94,95,96]. 

## 5. Stability of ACE-Inhibiting Fish-Derived Peptides to Gastrointestinal Digestion 

Small size peptides (mainly with two and up to six amino acids in the chain) cannot be substrates to any digestive proteases; therefore, they remain intact throughout gastrointestinal digestion. ACE inhibitory peptides must be absorbed from the intestine and reach the cardiovascular system in their original shape to hinder the activity of ACE [36]. Digestion stability of anti-ACE peptides to digestive enzymes were 95% for Ile-Trp, 100% for Leu-Trp, and 87% for salmon peptidic hydrolysate [51]. The effectivity of the two identified penta- and hexa-peptides of alcalase snakehead fish muscle sarcoplasmic protein hydrolysate (Leu-Tyr-Pro-Pro-Pro and Tyr-Ser-Met-Tyr-Pro-Pro) was tested in an in-vitro gastrointestinal (pepsin, pancreatin) and gastrointestinal + mucosal (lower intestinal peptidase) digestion model [54]. ACE inhibition, due to the presence of proline in the sequences was not affected by gastrointestinal digestion; however, gastrointestinal + mucosal digestion had non-significant negative effect [54,66]. Comparable effects were exhibited by a pH- and heat-stable penta-peptide (Val-Glu-Leu-Tyr-Pro) of cuttlefish hydrolysate showing resistance to the digestion of pepsin, trypsin, chymotrypsin or combination of all (IC_50_ 5.25–5.56 μM) [66]. 

Purified PHII-F3-3 of tuna-origin has not been tested for its resistance to digestive proteases, however, since Pro is situated at the C-terminus of the chain, therefore, this polypeptide might be well stable to the gastrointestinal digestion similar to the isolated penta- and hexa-peptide of snakehead’s muscle sarcoplasmic protein, Leu-Tyr-Pro-Pro-Pro, Tyr-Ser-Met-Tyr-Pro-Pro, etc. [54,57,66]. Val-Ala-Pro sequence of grass carp protein hydrolysate, with proline at the C-terminal, was also another example of a sequence that could resist pepsin and chymotrypsin, in an in-vitro digestive system model [45]. To improve the structure-activity relationship knowledge, identified peptides of dried bonito thermolysin hydrolysate and their fragments were synthesized slashing off amino acids from the C-terminal/N-terminal and or split in two fragmented products with the final fragment of the di- and tri-peptides by Yokoyama et al. [60]. The majority of the fragments were few to few hundred times less effective in comparison to the parent ACE inhibiting peptides [60]. Generally, fragmenting and splitting the synthesized parent peptide to di- and or tri-peptide improved the activity (Table 8) [60]. 

Gastrointestinal proteases’ (pepsin and pancreatin) digestion of cobia head protein hydrolysate surprisingly improved the activity of the fraction, MW < 3000 Da (67% abundant with peptides of molecular size 173–1749 Da), when hydrolyzed with pepsin and sequential pepsin/pancreatin digestion by 60% and 41%, respectively, in comparison to the pre-digestion condition (IC_50_ 0.24 mg mL^−1^), respectively [65]. The same fraction also resisted ACE hydrolysis (based on non-significant deviations of IC_50_ before and after pre-incubation with ACE) proving to be of an inhibitor type peptide [65]. Val-Ala-Pro sequence resisted ACE hydrolysis and proved to be of another inhibitor type drug rather than pro-drug type one [45]. Bioactive ACE inhibitor/antihypertensive dried bonito thermolysin hydrolysate peptides could be of inhibitor type or prodrug type, since when preincubated with ACE, IC_50_ values were significantly affected (Table 9) [77]. 

## 6. Conclusions and Possible Future Trends 

According to the results of studies, reviewed herein, the effectivity of the final biopeptide product is greatly affected by the extraction processes. The enzymatic hydrolysis (selection of enzymes, substrate: Enzyme ratio, degree of hydrolysis and process time), as well as isolation/purification techniques, are equally important for the production of the fish biopeptides with anti-ACE and anti-hypertensive potency. In this context, though practically not applicable to all fish types, such as bonito or tuna, alcalase commonly have been the enzyme of choice for many. Other types of enzymes are as well in common use for the efficient digestion of fish and fish-related products. For example, more potent biopeptides resulted from thermolysin and pepsin digestion of certain types of fish than alcalase digestion. 

Fish derived biopeptides whether from whole, byproducts and or even processed catch could be a natural source of ACE inhibitors/pro-drug type and antihypertensive peptides with no reported cytotoxicity. Accordingly, there is amply amount of data supporting the fact that these peptides could replace conventional synthetic drugs with similar potency and little to no adverse effects. These peptides are of various sizes and lengths; however, generally smaller MW peptides with only a few amino acids in their sequence have shown higher bioefficacy and bioavailability in comparison to the heavier MW polypeptides. Small biopeptides, in terms of MW and chain length, could better resist the digestive proteases and reach the target tissue intact to exhibit their health effect. In addition to size and chain length, certain factors in the structure, especially C-terminal, of a fish peptide are critical for ACE inhibition and antihypertensive effects. Presence of tyrosine, tryptophan, proline, phenylalanine and or positively charged amino acids next to hydrophobic amino acids would make the interaction with active sites of ACE (competitive mode of inhibition) and non-active sites of ACE (non-competitive mode of inhibition) possible. Another important feature could be the presence of proline at C-terminal so that the peptide could resist the gastrointestinal enzymes, pepsin, trypsin, and chymotrypsin for the reasons mentioned earlier. Many of the herein reviewed fish antihypertensive oligopeptides, especially di- and tri-peptides, may be suitable drug candidates considering their adsorption, distribution, metabolism, and excretion (ADME) characteristics. Drug-likeness of selected tri-, tetra- and penta-peptides, listed in Table 10, was predicted according to Lipinski’s rule of five using SwissADME web tool. However, it is noteworthy that Lipinski’s rules, mainly appropriate for oral dosage forms, are only prediction measures and many successful inhibitor drugs have shown to violate at least two of the rules. This may be the case for Leu-Lys-Pro-Asn-Met, a Bonito-extracted penta-peptide, violating two of the Lipinski’s rules yet being an effective inhibitor, marketed for controlling high blood pressure in tablet and capsule dosage forms (Table 10). 

In the light of this evidence, high blood pressure could be managed and treated by fish biopeptides effectively. Aforementioned health effects could not be solitary specific to grass carp, salmon, sardine or tilapia; hence, many other fish types may have similar effects, yet more research is warranted for a concrete supposition. However, successful discovery and design of natural ACE inhibitors necessitate more structure-function analysis of the blood pressure lowering fish-biopeptides. Examining the feasibility of industrial scale-up production ought to go hand in hand with the biopeptides’ incorporation into commercial products. Interestingly some of the anti-ACE fish-biopeptides have made their way through the nutraceutical and pharmaceutical industries and are the active ingredients of numerous natural health products (Table 11). These products are already marketed in North America, Europe and Asia in various forms (Table 11). To conclude, bioactive peptides of many fish sources could be good candidates for the development of natural health foodstuffs, nutraceutical and pharmaceutical products, however, more human studies and clinical trials are needed to better evaluate their bioefficacy and bioavailability in the target site and the subject. It is also of note that only about one third (12 out of 37) of the herein reviewed research articles utilized fishery byproducts, mainly frame, skin and scale, as substrates and starting raw material for the extraction of biologically active peptides. Nevertheless, it is expected that these and similar research projects make efficient use of the process byproducts and underutilized species in order to partially contribute to the targets of food security and greenhouse gas emission strategies. 

## Figures and Tables

**Table 1:** Definition and criteria of metabolic syndrome by different organizations.

Organization	Criteria for Cardiometabolic Syndrome
NCEP-ATP III	Dyslipidemia Central obesity Systemic arterial hypertension and hyperglycemia
IDF, EGIR, AACE	Central obesity Insulin resistance
WHO	Diabetes mellitus Insulin resistance any of two risk factors (obesity, hypertension, high triglycerides, reduced HDL-C level, or micro-albuminuria)

AACE: American Association for Clinical Endocrinology, EGIR: European Group for the study of Insulin Resistance, IDF: International Diabetes Federation, NCEP-ATP III: National Cholesterol Education Program Adult Treatment Panel III, WHO: World Health Organization.

**Table 2:** The cost of care for hypertensive patients with metabolic syndrome components in the European Community.

Country	Approximate Mean Annual Cost (Euros, 2008) Per Individual	Total Annual Cost-of-Illness (Million Euros, 2008)	Forecasted Total Annual Cost-of-Illness (Million Euros, 2020)
Germany	1750	24,427	38,955
Spain	1125	1900	5329
Italy	2000	4877	12,523

Adapted from Scholze et al. [7].

**Table 3:** Salmon di- and tri- peptides with identical counterpart sequences found in sources other than fish.

Peptide Properties				
Sequence	Mass (Da)	Hydrophobicity (Kcal mol^−1^)	Sources Other than Fish	Lowest IC_50_ (μM) Reported
**Dipeptides**				
DP	230.0900	+11.68	Marine	2.15
FL	278.1626	+4.94	Plant	1.33
FY	328.1419	+5.48	Animal, plant, marine, insect	1.67
GW	261.1111	+6.96	Animal, plant	30.00
IF	278.1626	+5.07	Animal, plant, insect	1.67
LF	278.1626	+4.94	Animal, plant	4.00
LW	317.1735	+4.56	Animal, plant, marine	2.50
PP	212.1158	+8.1	Animal, plant	-
VF	264.1470	+5.73	Animal, plant, marine, insect	2.70
VL	230.1626	+6.19	Plant	13.00
VP	214.1314	+7.58	Animal, plant	420.00
VW	303.1579	+5.35	Animal, plant, marine	1.10
YP	278.1263	+7.33	Animal, plant	720.00
**Tripeptides**				
AFL	349.1996	+5.44	Marine	63.80
IVF	377.2308	+4.61	Animal, plant	33.11
LVL	343.2464	+4.94	Animal, plant	12.30

Data extracted from reviews of Lee et al., Daskaya-Dikmen et al., Kumar et al., Iwaniak et al. and AHTPDB database [23,24,35,36].

**Table 4:** Physiochemical properties of ACE.

Properties	Values
Chemical Formula	C_6787_H_10263_N_1825_O_1930_S_44_
Number of amino acids	1306
Total number of atoms	20849
Molecular weight	149714.86 Da
Charge	Negative
Grand average of hydropathicity (GRAVY)	−0.398
Total no. of negatively charged residues	(Asp + Glu): 147
Total no. of positively charged residues	(Arg + Lys): 121

Properties of human ACE (P12821) were predicted by ProtParam.

**Table 5:** Fractionation, purification and characterization methods of fish anti-ACE and antihypertensive peptides.

Fish Name (Common, Scientific) and Part Used	Hydrolysis, Fractionation, Purification	Condition and Resin/Material	Characterization	References
Grass carp fresh whole	1—enzymatic hydrolysis 2—ultrafiltration (UF) 3—column chromatography, two-steps Reversed-phase high-performance liquid chromatography (RP-HPLC)	1—alcalase (degree of hydrolysis (DH): 17.25%) 2—molecular weight cut-off (MWCO) 10, 3 kDa 3—DA 201-C macroporous adsorption resin column, Jupiter and Luna C_18_ column	Automated protein/peptide sequencer, Dawn Eos multi-angle laser light scattering combined with gel permeation chromatography (GPC/MALLS), RP-HPLC (cation exchange resin column)	[43,45]
Grass carp (*Ctenopharyngodon idellus*) fresh scales	1—enzymatic hydrolysis 2—column chromatography	1—neutral protease AS1398 (DH: 16%) 2—DA 201-C macroporous adsorption resin	Amino acid analyzer, advanced protein purification system (TSK gel column)	[46]
Grass carp (*Ctenopharyngodon idellus*) fresh whole	1—enzymatic hydrolysis 2—UF 3—desalting	1—alcalase, neutrase 2—MWCO 10 kDa 3—mixed ion exchange resins (cation exchange resin (001 × 16, Na type) and anion exchange resin (D301-G, Cl type))	HPLC with gel permeation chromatography (TSK gel column), HPLC (cation exchange column)	[42]
Carp (Cyprinus carpio) fresh muscle tissue	1—ex-vivo digestion	1—human gastric and duodenal enzymes	Sodium dodecyl sulphate-polyacrylamide gel electrophoresis (SDS-PAGE)	[47]
Atlantic salmon (*Salmo salar*), Coho salmon (*Onchorhynchus kisutch*), Alaska pollack (*Theragra chalcogramma*), southern blue whiting (*Micromesistius australis*) fresh muscle tissues	1—enzymatic hydrolysis 2—UF 3—size exclusion chromatography	1—pepsin, pancreatin, thermolysin (*Bacillus thermoproteolyticus*), 2—MWCO 5 kDa 3—Bio-Gel P-2 column		[48]
Chub mackerel (*Scomber japonicus*) fresh whole fish followed by fermentation	1—aqueous extraction 2—ion-exchange chromatography	1—hot water 2—cation-exchange resin (TSK-Gel)	HPLC (COSMOSIL 5C_18_-AR column)	[49]
Salmon pectoral fin by-product	1—enzymatic hydrolysis 2—ion-exchange chromatography, size exclusion chromatography, RP-HPLC	1—alcalase, flavourzyme, neutrase, protamex, pepsin, trypsin (DH: 10%) 2—HiPrep 16/10 diethylaminoethyl (DEAE) fast flow (FF) ion-exchange column, Sephadex G-25 gel filtration column, octadecylsilyl (ODS) C_18_ column	Hybrid quadrupole- time-of-flight (TOF) LC/MS/MS mass spectrometer coupled with electrospray ionization (ESI) source	[44]
Chum salmon (*Oncorhynchus keta*) fresh muscle tissue	1—enzymatic hydrolysis 2—column chromatography, size exclusion chromatography, RP-HPLC	1—thermolysin 2—octadecyl silica gel column, Sephadex G-25 column, preparative ODS column	Protein sequencer after automated Edman degradation, amino acid analyzer, ESI-MS	[30,31]
Salmon (*Salmo salar*) trimmings	1—enzymatic hydrolysis 2—semi-preparative RP-HPLC	1—different preparations and 1, 2 and 4 h durations (alcalase 2.4 L (DH: 13.98–18.35%), alcalase 2.4 L + flavourzyme 500 L (DH: 15.15–16.02%), corlolase PP (DH: 21.83–24.34%), PROMOD 144MG (DH: 20.34–22.11%) 2—C_18_ semi-preparative column	SDS-PAGE, RP-HPLC, gel permeation HPLC, ultra-performance liquid chromatography (UPLC)-MS/MS (Peptide XB-C_18_ column)	[50]
Pink salmon (*Oncorhynchus gorbuscha*) muscle	1—enzymatic hydrolysis 2—column chromatography, ion-exchange chromatography	1—papain 2—silica gel, cation exchange resin (CG50-type 1)	Nuclear magnetic resonance (NMR), ESI-MS, LC/MS (XTerra MS C_18_ column), HPLC (C_18_ PA-A column)	[51]
Sardine muscle	1—alkali solubilization 2—enzymatic hydrolysis 3—RP-HPLC	1—20% NaOH 2—*Bacillus licheniformis* alkaline protease 3—YMC ODS-AQ column		[52]
Tilapia (*Oreochromis niloticus*) fresh whole	1—enzymatic hydrolysis 2—UF 3— fast protein liquid chromatography (FPLC), ion exchange chromatography, size exclusion chromatography	1—*Virgibacillus halodenitrificans* SK1-3-7 proteinases (DH: 48%) 2—MWCO 30, 5 kDa 3—DEAE-Sephacel anion exchange column, carboxymethyl (CM)-Sepharose cation exchange column, Superdex peptide 10/300 GL size exclusion column	LC-MS/MS (LC ( (Acclaim PepMap 100 C_18_ nanocolumn) coupled to ESI-Ion Trap MS with electrospray)	[53]
Snakehead fish muscle	1—enzymatic hydrolysis 2—gel chromatography, RP-HPLC	1—flavorzyme, neutrase, proteinase k, papain, alcalase 2—polyacrylamide Bio-Gel P-2 column, C_18_ column	LC-ESI-MS (RP C_18_ PepMap 100 column in HPLC connected to micro-TOF-Q-MS) and Tandem MS analysis	[54]
Seaweed pipefish (*Syngnathus schlegeli*) fresh muscle	1—enzymatic hydrolysis 2—FPLC, RP-HPLC	1—papain, alcalase, neutrase, pronase, pepsin, trypsin 2—HiPrep 16/10 DEAE FF ion exchange column, Primesphere C_18_ column	Q-TOF-MS coupled with ESI	[55]
Sea bream fresh scales	1—enzymatic hydrolysis 2—size exclusion chromatography, ion-exchange chromatography, RP-HPLC	1—enzyme L 2—Sephadex LH-20 gel filtration column, UNO Q-1 column, Superdex Peptide HR 10/30 gel filtration column, Sephasil Peptide C_18_ column	Protein sequencer after automated Edman degradation	[56]
Tuna frame	1—enzymatic hydrolysis 2—UF 3—ion-exchange chromatography, RP-HPLC	1—alcalase, α-chymotrypsin, papain, pepsin, neutrase, trypsin 2—MWCO 10, 5, 1 kDa 3—Hiprep 16/10 DEAE FF anion exchange column, Primesphere 10 C_18_ column, Synchropak RPP-100 analytical column	Q-TOF-MS coupled with ESI	[57]
Sardine muscle	1—enzymatic hydrolysis 2—RP-HPLC	1—*Bacillus licheniformis* alkaline protease (different enzyme: Product ratio and 1, 17 and 24 h duration) 2—YMC ODS-AQ column	Asahipak GS-320 gel filtration chromatography	[58,59]
Bonito dehydrated whole	1—enzymatic hydrolysis 2—HPLC	1—pepsin, trypsin, chymotrypsin, thermolysin 2—YMC ODS-AQ column, phenyl silica column, cyanopropyl silica column, ODS C_18_ column	Protein sequencer	[60]
Zebra blenny (*Salaria basilisca*) fresh muscle	1—enzymatic hydrolysis	1—crude alkaline protease extracts extracted from homogenized viscera of zebra blenny, sardinella and smooth hound		[61]
Thornback ray (*Raja clavata*) muscle	1—enzymatic hydrolysis	1—alcalase 2.4L (DH: 22%), neutrase 0.5L (DH: 11%), *B. subtilis* A26 (DH: 18%), *R. clavata* crude alkaline protease (DH: 15%)	RP-HPLC (symmetry C_18_ column), MALDI-TOF/TOF	[62]
Bigeye tuna dark muscle	1—enzymatic hydrolysis 2—UF 3—ion-exchange chromatography, RP-HPLC	1—alcalase (DH: 67.22%), α-chymotrypsin (DH: 77.71%), neutrase (DH: 80.93%), papain (DH: 74.45%), pepsin (DH: 78.72%), trypsin (DH: 46.28%) 2—MWCO 3 kDa 3—HiPrep 16/10 DEAE FF ion-exchange column, Primesphere ODS C_18_ column, Synchropak RPP-100 RP-HPLC analytical column	Q-TOF-MS coupled with ESI	[63]
Yellowfin sole (*Limanda aspera*) fresh frame	1—enzymatic hydrolysis 2—UF 3—ion-exchange chromatography, gel permeation chromatography, RP-HPLC	1—α-chymotrypsin 2—MWCO 30, 10, 5 kDa 3—SP-Sephadex C-25 ion-exchange column, OHpak SB-803 HQ gel permeation HPLC column, SP Nucleosil 100-7 C_18_ RP semi-prep column, Zorbax SB C_18_ RP analytical column	Gel permeation chromatography (OHpak SB-803 HQ gel permeation HPLC column)	[64]
Cobia (*Rachycentron canadum*) fresh head	1—enzymatic hydrolysis 2—UF	1—papain, 2—MWCO 8, 5, 3 kDa	High performance size exclusion chromatography (Protein-Pak 60 column)	[65]
Cuttlefish (*Sepia officinalis*) fresh muscle	1—enzymatic hydrolysis 2—gel filtration column chromatography, RP-HPLC	1—*Bacillus mojavensis* A21 proteases (DH: 16%), cuttlefish hepatopancreas proteases (DH: 8%) 2—Sephadex G-25 gel filtration column, Vydac C_18_ column	ESI–MS, MS/MS	[66]
Skate (*Okamejei kenojei*) fresh skin	1—alkaline and aqueous gelatin extraction 2—enzymatic hydrolysis 3—UF 4—FPLC, gel filtration chromatography	1—1% Ca(OH)_2_, water 2—alcalase, flavourzyme, protamex, neutrase, protease, α-chymotrypsin 3—MWCO 1 kDa 4—HiPrep 16/10 DEAE FF anion-exchange column, Superdex Peptide 10/300 GL gel filtration column	Q-TOF-MS coupled with ESI	[39,67]
Alaska Pollack (*Theragra chalcogramma*) frame	1—enzymatic hydrolysis 2—UF 3—ion-exchange chromatography, size exclusion chromatography, RP-HPLC	1—pepsin 2—MWCO 30, 10, 5, 3, 1 kDa 3—SP-Sephadex C-25 ion-exchange column, Sephadex G-25 gel filtration column, Capcell Pak C_18_ UG-120 column	Protein sequencer after automated Edman degradation,	[68]
Pacific cod (*Gadus macrocephalus*) fresh skin	1—alkaline and aqueous gelatin extraction 2—enzymatic hydrolysis 3—FPLC, HPLC, RP-HPLC	1—1% Ca(OH)_2_, water 2—gastrointestinal endopeptidases (pepsin, trypsin and α-chymotrypsin) 3—HiPrep 16/10 DEAE FF anion exchange column, Primesphere 10 C_18_ column, YMC-Pack Pro C_18_ column	Q-TOF-MS coupled with ESI	[69]
Brownstripe red snapper (*Lutjanus vitta*) fresh flesh	1—enzymatic hydrolysis (one step and two steps)	1—one step: Proteases from pyloric caeca of brownstripe red snapper (DH: 20–40%), flavourzyme (DH: 20–40%), alcalase (DH: 20–40%), two steps: Hydrolysates with 40% DH (durations 1, 2, 3, 5 h)	Amino acid analyzer	[70]
Tilapia (*Oreochromis niloticus*) fresh white muscle	1—alkaline solubilization 2—enzymatic hydrolysis 3-UF	1—2 N NaOH 2—flavourzyme (DH: 7.5–25%), cryotin-F (DH: 7.5–25%) 3—MWCO 30, 10 kDa	SDS–PAGE	[71]
Stone fish (*Actinopyga lecanora*) fresh whole (excluding internal organs)	1—enzymatic hydrolysis 2—RP-HPLC, isoelectric focusing (IEF)- electrophoresis	1—bromelain 2—semi preparative C_18_ column	Q-TOF LC/MS	[72]
Pacific cod (*Gadus macrocephalus*) fresh skin	1—alkaline and aqueous gelatin extraction 2—enzymatic hydrolysis 3—UF 4—FPLC, gel filtration chromatography, RP-HPLC	1—1% Ca(OH)_2_, water 2—pepsin, papain, α-chymotrypsin, trypsin, neutrase, alcalase 3—MWCO 10, 5, 1 kDa 4—HiPrep 16/10 DEAE FF anion exchange column, Superdex Peptide 10/300 GL gel filtration column	Q-TOF LC/MS/MS coupled with ESI	[73]

**Table 6:** Biological activity of fish and fish by-products’ derived peptides on hypertension.

Fish Name	Anti-ACE and Antihypertensive Effects	Sequence and Molecular Mass	References
Grass carp	- ACE inhibition, hydrolysate; IC_50_ 0.872 mg mL^−1^, < 3 kDa; IC_50_ 0.308 mg mL^−1^, < 3 kDa fraction 6; IC_50_ 0.00553 mg mL^−1^, F6-I; IC_50_ 0.00534 mg mL^−1^, VAP; IC_50_ 0.00538 mg mL^−1^	F6-I rich in VAP 285 Da	[45]
	- ACE inhibition, < 3 kDa; IC_50_ 0.23 mg mL^−1^, - Antihypertension activity, single oral administration of 100 mg kg^−1^ BW ~30 mmHg maximal drop of systolic blood pressure (SBP) at 6 h in SHR dose/time dependent and non-significant effect in normotensive rats, long-term study, 100 mg kg^−1^ BW ~20 mmHg maximal drop of SBP at four weeks in SHR and non-significant effect in normotensive rats	Fraction < 3 kDa rich in Leu, Asp, Phe, Gly, Pro, hydrophobic amino acids (AAs) at C-terminal (44.15%), 1022–1228 Da (56.6%), 725–834 Da (43.3%)	[43]
Grass carp (*C. idellus*)	- ACE inhibition, hydrolysate; IC_50_ 1.66 mg mL^−1^, after ethanol gradient elution (v/v), 20% ETOH; IC_50_ 0.48mg mL^−1^, 40% ETOH; IC_50_ 0.39 mg mL^−1^, 60% ETOH; IC_50_ 0.25mg mL^−1^, 80% ETOH; IC_50_ 0.13 mg mL^−1^, % ACE inhibition at 2.5 mg mL^−1^ hydrolysate; ~70%, after ethanol gradient elution (v/v), 20% ETOH; ~75%, 40% ETOH; ~85%, 60% ETOH; ~90%, 80% ETOH; >95% dose-dependent effects	Hydrolysate and fractions rich in hydrophobic AAs (31–38%) and Pro, Gly	[46]
Grass carp (*C. idellus*)	- ACE inhibition, hydrolysate; IC_50_ 0.692 mg mL^−1^, < 10 kDa; IC_50_ 0.272 mg mL^−1^, desalted < 10 kDa; IC_50_ 0.105 mg mL^−1^, - Antihypertensive activity, 100 mg kg^−1^ BW 43 mm Hg maximal SBP drop in SHR	Desalted < 10 kDa rich in < 1000 Da (190–500 Da (59.2%), 500–1000 Da (32.3%))	[42]
Carp (C. carpio)	- ACE inhibition, two-hour gastric and one-hour duodenal digested fraction; IC_50_ 1.90 mg mL^−1^, two-hour gastric digested fraction; IC_50_ 9.26 mg mL^−1^		[47]
Atlantic salmon (*S. salar*), Coho salmon (*O. kisutch*), Alaska pollack (*T. chalcogramma*), southern blue whiting (*M. australis*)	- ACE inhibition, Atlantic salmon; IC_50_ 7.91 mg mL^−1^, Coho salmon; IC_50_ 4.66 mg mL^−1^, Alaska pollack; IC_50_ 3.41 mg mL^−1^, southern blue whiting; IC_50_ 3.62 mg mL^−1^	Hydrolysates and fractions rich in anserine	[48]
Chub mackerel (*S. japonicus*)	- ACE inhibition, hot-water extract of raw mackerel; IC_50_ 0.2 mg mL^−1^, hot-water extract of fermented mackerel; IC_50_ 0.06 mg mL^−1^, - Antihypertension activity, administration of 10 mg kg^−1^ BW, 27 mm Hg maximal SBP drop in SHR at 4 h		[49]
Salmon	- ACE inhibition, alcalase hydrolysate; IC_50_ 0.356 mg mL^−1^, P1; IC_50_ 0.00912 mg mL^−1^, non- competitive mode, P2; IC_50_ 0.01372 mg mL^−1^, mixed mode, P4; IC_50_ 0.00679 mg mL^−1^, mixed mode	P1 (VWDPPKFD ~ 1003 Da), P2 (FEDYVPLSCF ~ 1219 Da), P4 (FNVPLYE ~ 881 Da)	[44]
Chum salmon (*O. keta*)	- ACE inhibition, hydrolysate; IC_50_ 0.0381 mg mL^−1^, 10% ethanol fraction O-2; IC_50_ of 0.0286 mg mL^−1^, positive fraction of gel filtration chromatography S-4; IC_50_ of 0.0215 mg mL^−1^, FL; IC_50_ of 13.6 μM, non-competitive inhibition mode MW; IC_50_ of 9.8 μM, non-competitive inhibition mode	Rich in FL 278 Da	[30,31]
Salmon (*S. salar*)	- ACE inhibition, protein extract; IC_50_ 3.68 mg mL^−1^, alcalase hydrolysate; IC_50_ 0.74 mg mL^−1^, alcalase + flavourzyme hydrolysate; IC_50_ 0.94 mg mL^−1^, Corolase hydrolysate; IC_50_ 0.97 mg mL^−1^, PROMOD hydrolysate; IC_50_ 1.35 mg mL^−1^, FF; IC_50_ 59.15 μM, F; IC_50_ 0.15 μM, Y; IC_50_ 132.84 μM, YP; IC_50_ 5.21 μM	Two selected corolase fraction rich in GPAV, VP, VC, YP, FF, PP, DP, I/LD, I/LH, W, L/I, F, Y	[50]
Pink Salmon (*O. gorbuscha*)	- ACE inhibition, hydrolysate; IC_50_ 79 µg mL^−1^, IW; IC_50_ 0.38 µg mL^−1^, LW; IC_50_ 2.2 µg mL^−1^, % ACE inhibition, 20 µg mL^−1^ hydrolysate; 26%, aqueous fraction; 12%, 1-butanol soluble fraction; 51%, % ACE inhibition of sub-fractions of butanol-soluble fraction; 20 µg mL^−1^ 1^st^-silica gel column fractions (Fr.1-3); ≤ 71%, 6 µg mL^−1^ 2^nd^-silica gel column fractions (Fr.2-2); ≤ 48%, - Antihypertensive activity, single oral administration of 30 mg kg^−1^ BW salmon peptide, 20 mm Hg maximal SBP drop at 2 h in SHR, long-term administration of 1.0 g kg^−1^ BW d^−1^ of the salmon peptide; 5 mm Hg maximal SBP drop at 4-weeks in SHR	Ion exchange column fraction (Fr.3-5) rich in di-peptides (VW, GW, MW, LW, WA, WM, FL, LF, VL, LI, IL, VF, IF, YF, FY) and tri-peptides (VIF, IVF, FVL, AFL, LVL, VIL, IVL, FIA, YLV, IVW)	[51]
Sardine	- Antihypertensive activity, administration of 3 mg VY 9.7- and 5.3-mm Hg systolic/diastolic blood pressure (SBP/DBP) drop at 1 week, 9.3- and 5.2-mm Hg SBP/DBP drop at four weeks	VY 280 Da	[52]
Tilapia (*O. niloticus*)	- % ACE inhibition, hydrolysate; 85%, > 30 kDa; 51%, 5–30 kDa; 65%, < 5 kDa; 88%, fractions of < 5 kDa; DEAE-Sephacel; 59%, CM-Sepharose; 38%, Superdex peptide 10/300 GL; 35%, crude hydrolysate; IC_50_ 0.54 mg mL^−1^, Superdex peptide 10/300 GL-P-I; IC_50_ 0.15 mg mL^−^^1^ non-competitive mode, MILLLFR; IC_50_ 0.12 µM, LNLQDF; IC_50_ 0.51 µM, LNLQDFR; IC_50_ 0.85 µM	Superdex peptide 10/300 GL-P-I rich in MILLLFR 905.83 Da, LNLQDFR 905.66 Da, KHQDFF 821.41 Da, KPLLLMQLLLLFR 1526.64 Da, QNLLNYR 920.80 Da, QDLLFR 791.64 Da, NLGALLFR 903.49 Da, NELLLFR 904.60 Da, ELLGFV 677.49 Da, AHLLLL 679.49 Da	[53]
Snakehead fish	- ACE inhibition, crude sarcoplasmic protein; IC_50_ 3.34 mg mL^−1^, hydrolysate of papain; IC_50_ 0.182 mg mL^−1^, neutrase; IC_50_ 0.152 mg mL^−1^, flavourzyme; IC_50_ 0.149 mg mL^−1^, proteinase K; IC_50_ 0.137 mg mL^−1^, alcalase; IC_50_ 0.038 mg mL^−1^, post digestion LYPPP; IC_50_ 1.3 μM, YSMYPP; IC_50_ 2.8 μM	Alcalase hydrolysate rich in LYPPP 585 Da, YSMYPP 756 Da	[54]
Seaweed pipefish (*S. schlegeli*)	- % ACE inhibition, alcalase; 86%, - ACE inhibition, alcalase hydrolysate F3; IC_50_ 0.068 mg mL^−1^, F3-II; IC_50_ 0.62 mg mL^−1^, F3-III; IC_50_ 1.44 mg mL^−1^	Alcalase hydrolysate rich in F3-II; TFPHGP 744 Da, F3-III; HWTTQR 917 Da	[55]
Sea bream	- ACE inhibition, hydrolysate; IC_50_ 0.57 mg mL^−1^, fractions III; IC_50_ 0.096 mg mL^−1^, III-1; IC_50_ 0.052 mg mL^−1^, III-2; IC_50_ 0.080 mg mL^−1^, III-1C; IC_50_ 0.027 mg mL^−1^, III-1C-A; IC_50_ 0.063 mg mL^−1^, III-1C-B; IC_50_ 0.005 mg mL^−1^, III-1C-C; IC_50_ 0.16 mg mL^−1^, III-1C-D; IC_50_ 0.003 mg mL^−1^, - Antihypertensive activity, administration of 300 mg kg^−1^ BW d^−1^ hydrolysate significantly lowered blood pressure in SHR	Fractions rich in III-1C-A; GY 238 Da, III-1C-B; VY 280 Da, III-1C-C; GF 222 Da, III-1C-D; VIY 393 Da	[56]
Tuna	- % ACE inhibition, 2 mg mL^−1^ pepsin hydrolysate (PH); 88.2%, 1 mg mL^−1^ PHI (MW <1 kDa); ≥80%, 1 mg mL^−1^ PHII (MW 1–5 kDa); 91.6%, 1 mg mL^−1^ PHIII (MW 5–10 kDa); 89.4%, 0.2 mg mL^−1^ PHII-F3; 76.4%, 0.1 mg mL^−1^ PHII-F3-3; 86.5%, - ACE inhibition, pure peptide of PHII-F3-3; IC_50_ 11.28 μM, non-competitive inhibition mode - Antihypertensive activity, single oral administration of 10 mg kg^−1^ BW pure PHII-F3-3; 21 mmHg maximal SBP drop in SHR at 6 h	PHII-F3-3 rich in GDLGKTTTVSNWSPPKYKDTP 2482 Da	[57]
Sardine	- ACE inhibition, 1–2 wt%, 1 h; IC_50_ 0.24 mg mL^−1^, 0.1–0.5 wt%, 24 h; IC_50_ 0.25-0.26 mg mL^−1^, 0.3 wt%, 17 h; IC_50_ 0.26 mg mL^−1^, A-I (0.3 wt%, 17 h and 1 wt%, 1 h); IC_50_ 0.26 mg mL^−1^, A-I-Y1; IC_50_ 1.222 mg mL^−1^, A-I-Y2; IC_50_ 0.015 mg mL^−1^, A-I-Y3; IC_50_ 0.078 mg mL^−1^, A-I-Y4; IC_50_ 0.041 mg mL^−1^	A-I (0.3 wt%, 17 h and 1 wt%, 1 h) in decreasing order rich in AAs Glu > Asp > Lys > Leu 250–1000 Da	[58]
	- ACE inhibition, Hydrolysate; IC_50_ 62.4 mg mL^−1^, - Antihypertensive activity, single oral administration of 1 g kg^−1^ BW d^−1^; 13 mmHg maximal SBP drop in stroke-prone SHR at four weeks		[59]
Bonito	- ACE inhibition, thermolysin; IC_50_ 29 µg mL^−1^, trypsin; IC_50_ 161 µg mL^−1^, pepsin; IC_50_ 47 µg mL^−1^, chymotrypsin; IC_50_ 117 µg mL^−1^, trypsin/chymotrypsin; IC_50_ 175 µg mL^−1^, further digested peptic hydrolysate; trypsin; IC_50_ 65 µg mL^−1^, chymotrypsin; IC_50_ 41 µg mL^−1^, trypsin/chymotrypsin; IC_50_ 38 µg mL^−1^, further digested thermolysin hydrolysate; pepsin; IC_50_ 22 µg mL^−1^, pepsin → trypsin; IC_50_ 26 µg mL^−1^, pepsin → chymotrypsin; IC_50_ 29 µg mL^−1^, pepsin → trypsin/chymotrypsin; 26 IC_50_ of µg mL^−1^, IY; 3.7 μM, IWHHT; 5.1 μM, IVGRPRHQG; 6.2 μM, ALPHA; 10 μM, FQP; 12 μM, LKPNM; 17 μM, IKPLNY; 43 μM, DYGLYP; 62 mΜ - Antihypertensive activity, administration of 10 mg kg^−1^ BW of IKP/IVGRPRHQG significantly inhibited the elevation of blood pressure in normotensive rat	Thermolysin hydrolysate rich in; IKPLNY 746 Da, IVGRPRHQG 1019 Da, IWHHT 692 Da, ALPHA 507 Da, LKPNM 601 Da, IY 294 Da, FQP 390 Da, DYGLYP 726 Da	[60]
Zebra blenny (*S. basilisca*)	- ACE inhibition, hydrolysate of zebra blenny protease; IC_50_ 93.6 µg mL^−1^, smooth hound protease; IC_50_ 130 µg mL^−1^, sardinella protease; IC_50_ 182 µg mL^−1^ dose-dependent inhibition, - in-vivo % ACE inhibition in serum of surviving diabetic rats; zebra blenny protease; 52.8%, smooth hound protease; 53%, sardinella protease; 56%, - Antihypertensive activity, adverse effect on diabetic (alloxan-induced) rats		[61]
Thornback ray (*R. clavata*)	- % ACE inhibition at 5 mg mL^−1^, crude muscle protein; 15%, hydrolysate of alcalase; 84%, neutrase; 87%, *B. subtilis* A26; 87%, *R. clavata* crude alkaline protease; 51%	Crude mix and hydrolysates rich in Glu, Gly, Pro	[62]
Bigeye tuna	- ACE inhibition, purified PIII-2; IC_50_ 21.6 µM, non-competitive inhibition mode, - % ACE inhibition at 2 mg mL^−1^, hydrolysate of alcalase; 48%, α-chymotrypsin; 57%, neutrase; 64%, papain; 20%, pepsin; 81%, trypsin; 36%, pepsin hydrolysate fraction PIII; 78%, PIII-2; ~80%, - Antihypertensive activity, oral administration of 10 mg kg^−1^ BW ~17 mm Hg maximal SBP drop in SHR at 3 h and 6 h	PIII-2 rich in WPEAAELMMEVDP 1581 Da	[63]
Yellowfin sole (*L. aspera*)	- % ACE inhibition; 30–10 kDa; 47.6%, 10–5 kDa; 34.5%, <5 kDa; 68.8%, - ACE inhibition, <5 kDa; IC_50_ 0.883 mg mL^−1^ (22.3 μM), fractions of < 5 kDa; cation exchange chromatography; IC_50_ 0.210 mg mL^−1^, gel permeation chromatography; IC_50_ 0.093 mg mL^−1^, 1^st^ RP-HPLC; IC_50_ 0.056 mg mL^−1^, 2^nd^ RP-HPLC; IC_50_ 0.029 mg mL^−1^, undigested protein; significant ACE inhibition at 250 µM, 500 mM, non-competitively, - Antihypertensive activity, single oral administration of 10 mg kg^−1^ BW 22 mmHg maximal SBP drop in SHR at 3 h	Undigested protein rich in MIFPGAGGPEL 1.2 kDa and <5 kDa fraction rich in hydrophobic AAs	[64]
Cobia (*R. canadum*)	- ACE inhibition, hydrolysate; IC_50_ 0.57 mg mL^−1^, >8 kDa; IC_50_ 1.06 mg mL^−1^, 8–5 kDa; IC_50_ 0.73 mg mL^−1^, 5–3 kDa; IC_50_ 0.36 mg mL^−1^, <3 kDa; IC_50_ 0.24 mg mL^−1^, - Antihypertensive activity, oral administration of 150, 600, 1200 mg kg^−1^ BW significantly lowered SBP in SHR dose-dependently at 2–8 h, 1200 mg kg^−1^ BW, 57 mmHg maximal SBP drop in SHR at 4 h	<3 kDa fraction; 67% (1749–173 Da), 11% (2831–1747 Da), 16% (7875–2831 Da)	[65]
Cuttlefish (*S. officinalis*)	- ACE inhibition; A21 proteases hydrolysate; IC_50_ 1.12 mg mL^−1^, Cuttlefish proteases hydrolysate; IC_50_ 1.19 mg mL^−1^, Peptides of A21 proteases hydrolysate: SFHPYFSY; IC_50_ 82.71 μM AFVGYVLP; IC_50_ 18.02 μM KNGDGY; IC_50_ 51.63 μM STHGVW; IC_50_ 19.30 μM RSIKGF; IC_50_ 32.74 μM GS; IC_50_ 1156.3 μM Peptides of cuttlefish proteases hydrolysate: GIHETTY; IC_50_ 25.66 μM EKSYELP; IC_50_ 14.41 μM VELYP; IC_50_ 5.22 μM - Antihypertensive activity, oral administration of VELYP 10 mg kg^−1^ BW d^−1^ decreased SBP and DBP at 2–8 h post-administration non-cytotoxically (20 mmHg maximal SBP drop) in SHR	Hydrolysates rich in SFHPYFSY 1047.1 Da, AFVGYVLP 865.4 Da, KNGDGY 653.2 Da, STHGVW 663.1 Da, RSIKGF 665.0 Da, GS 163.0 Da, GIHETTY 820.3 Da, EKSYELP 865.1 Da, VELYP 620.1 Da	[66]
Skate (*O. kenojei*)	- % ACE inhibition, 2 mg mL^−1^ Alcalase gelatin hydrolysate; 72.8%, 1 mg mL^−1^ alcalase/protease gelatin hydrolysate < 1 kDa (SAP); 86%, 100 µg mL^−1^ SAP-I; 73%, 100 µg mL^−1^ SAP-I3; 85% MVGSAPGVL; IC_50_ 3.09 μM, LGPLGHQ; IC_50_ 4.22 μM	SAP-I3 rich in MVGSAPGVL 829 Da, LGPLGHQ 720 Da	[67]
	- Antihypertensive activity, oral administration of 1 g SAP kg^−1^ BW d^−1^, 20 days, 127.2 mmHg maximal SBP drop, 77.6 mmHg maximal DBP drop, 94.2 mmHg maximal mean blood pressure drop at day 20, in SHR	SAP rich in Lys 31% > Gly 16% > Glu 7%, SAPI-3 rich in	[39]
Alaska Pollack (*T. chalcogramma*)	- ACE inhibition <1 kDa; IC_50_ 0.457 mg mL^−1^ SP-Sephadex C-25; IC_50_ 0.11 mg mL^−1^ Sephadex G-25; IC_50_ 0.066 mg mL^−1^ 1^st^ RP-HPLC; IC_50_ 0.023 mg mL^−1^ 2^nd^ RP-HPLC; IC_50_ 0.013 mg mL^−1^ FGASTRGA; IC_50_ 14.7 μM	2^nd^ RP-HPLC fraction rich in FGASTRGA 765 Da	[68]
Pacific cod (*G. macrocephalus*)	- ACE inhibition 100 µg mL^−1^ Hydrolysate; 60.40%, FPLC; ≤71.81%, HPLC; ≤77.85%, LLMLDNDLPP; IC_50_ 35.7 μM	HPLC fraction rich in LLMLDNDLPP 1301 Da	[69]
Brownstripe red snapper (*Lutjanus vitta*)	- % ACE inhibition ~ ≤33%	Hydrolysates rich in hydrophobic AAs ~ 44% > charged AAs ~ 43% > polar AAs 13%	[70]
Tilapia (*Oreochromis niloticus*)	- % ACE inhibition, 0.2% w/v protein cryotin hydrolysates 62–71%, flavourzyme hydrolysates 66–73%	MW range 3.5–110 kDa	[71]
Stone fish (*Actinopyga lecanora*)	- % ACE inhibition, RP-HPLC fractions; 43.50%, IEF electrophoresis sub-fractions; 69.21%, ALGPQFY; IC_50_ 0.012 mM, KVPPKA; IC_50_ 0.980 mM, LAPPTM; IC_50_ 1.310 mM, EVLIQ; IC_50_ 1.440 mM, EHPVL; IC_50_ 1.680 mM	ALGPQFY 794.44 Da, KVPPKA 638.88 Da, LAPPTM 628.85 Da, EVLIQ 600.77 Da, EHPVL 593.74 Da	[72]
Pacific cod (*Gadus macrocephalus*)	- % ACE inhibition, 1 mg mL^−1^ pepsin hydrolysate; 91%, 500 µg mL^−1^ pepsin hydrolysate < 1 kDa; 70%, 250 µg mL^−1^ FPLC fraction of < 1 kDa (F1); 81%, 100 µg mL^−1^ gel column fraction of F1; 96%, GASSGMPG; IC_50_ 6.9 μM, LAYA; IC_50_ 14.5 μM	GASSGMPG 662 Da, LAYA 436 Da	[73]
Lean (white) fish	- Antihypertensive activity, 4 meals/week lean fish, 8-week controlled study, 5 mm Hg maximal SBP drop (3.5 ± 3.2% decreased), 4 mm Hg maximal DBP drop (4.6 ± 3.6% decreased) in coronary heart disease patients using multiple medications		[74]
Purified fish protein (salmon, tuna, cod, mix of white fishes) (94% protein)	- Antihypertensive activity, 20.4 g kg^−1^ BW d^−1^ (20% protein diet), 2-months controlled study, 30.9 mm Hg maximal SBP drop (14% decrease compare to casein-diet) in SHR at 4, 6, 7 wk up to 2-months	Diet rich in Glu 13% > Gly 12.3% > Asp 8% > Lys 7% > Ala 6.9% > Arg 6.5%	[75,76]
Bonito, LKP from muscle	- ACE inhibition, LKPNM; IC_50_ 2.4 μM, LKP; IC_50_ 0.3 μM, - Antihypertensive activity, intravenous injection of 100 μg kg^−1^ of LKPNM; 30 mm Hg maximal SBP drop, 30 μg kg^−1^ of LKP; 50 mm Hg maximal SBP drop dose-dependently in SHR oral administration of 10.5 mg kg^−1^ LKPNM at 4 h and 4.2 mg kg^−1^ LKP at 2 h; 10 mm Hg maximal SBP drop in SHR		[77]

**Table 7:** Structures of selected fish-extracted antihypertensive and ACE inhibitory oligopeptides.

Peptide’s Primary Structure	Peptide Sequence IC_50_ Mass (Da) Isoelectric point Net Charge and Hydrophobicity (Kcal mol^−1^)	Peptide’s Primary Structure	Peptide Sequence IC_50_ Mass (Da) Isoelectric Point Net Charge and Hydrophobicity (Kcal mol^−1^)
**Penta-peptides**			
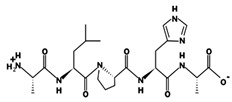	ALPHA 10 μM [60] 507.2798 7.95 0 +10.12	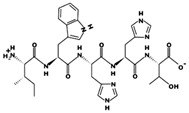	IWHHT 5.1 μM [60] 692.3386 8.05 0 +9.60
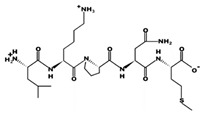	LKPNM 17 μM [60] 601.3248 10.14 +1 +9.77	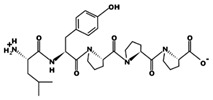	LYPPP 1.3 μM [54] 585.3153 5.22 0 +6.36
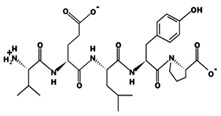	VELYP 5.22 μM [66] 619.3207 3.01 −1 +9.25	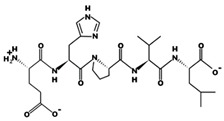	EHPVL 1.680 mM [72] 593.3164 5.06 −1 +12.29
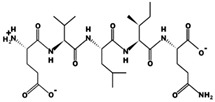	EVLIQ 1.440 mM [72] 600.3472 3.12 −1 +9.47		
**Hexa-peptides**			
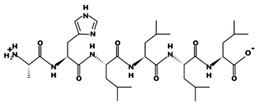	AHLLLL - 678.4416 7.95 0 +5.73	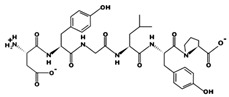	DYGLYP 62 mΜ [60] 349.1633 5.48 0 +7.83
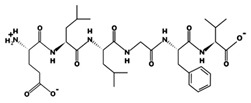	ELLGFV - 676.3784 3.23 −1 +8.01	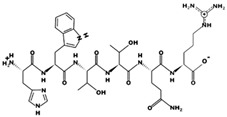	HWTTQR 1.74 mM [55] 827.4028 10.91 +1 +11.22
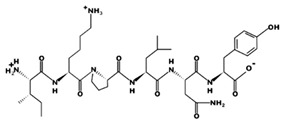	IKPLNY 43 μM [60] 746.4314 9.79 +1 +8.61	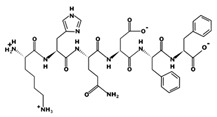	KHQDFF - 820.3857 7.55 0 +14.02
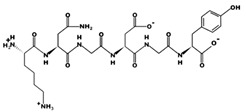	KNGDGY 51.63 μM [66] 652.2808 6.42 0 +16.78	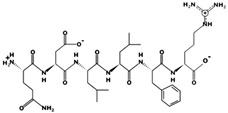	QDLLFR - 790.4325 6.48 0 +9.91
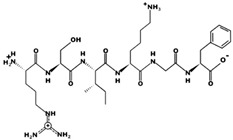	RSIKGF 32.74 μM [66] 706.4115 11.52 +2 +11.29	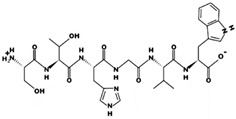	STHGVW 19.30 μM [66] 685.3175 7.63 0 +9.54
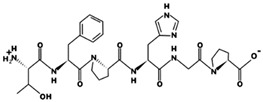	TFPHGP 947.56 μM [55] 654.3117 7.57 0 +10.20	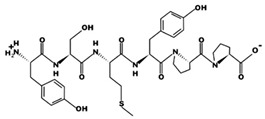	YSMYPP 2.8 μM [54] 756.3142 5.17 0 +6.55
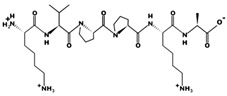	KVPPKA 0.980 mM [72] 638.4104 10.57 +2 +13.82	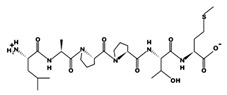	LAPPTM 1.310 mM [72] 628.3244 5.40 0 +7.01
**Hepta-peptides**			
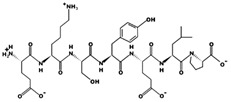	EKSYELP 14.41 μM [66] 864.4215 4.08 −1 +16.60	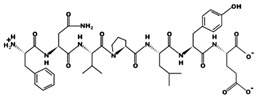	FNVPLYE 7.71 μM [44] 880.4317 3.09 −1 +8.39
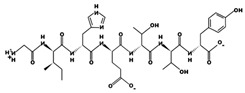	GIHETTY 25.66 μM [66] 819.3751 5.06 −1 +13.68	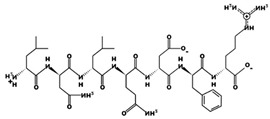	LNLQDFR 0.85 µM [53] 904.4753 6.74 0 +10.76
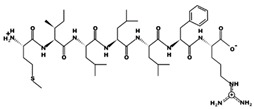	MILLLFR 0.12 µM 904.5552 [53] 10.88 +1 +2.46	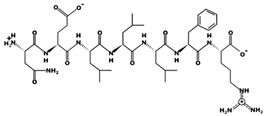	NELLLFR - 903.5163 6.38 0 +8.73
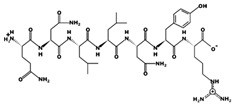	QNLLNYR - 919.4862 9.60 +1 +8.97	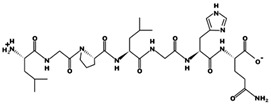	LGPLGHQ 4.22 μM [67] 720.3908 7.89 0 +10.94
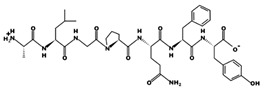	ALGPQFY 0.012 mM [72] 794.3951 5.46 0 +6.79		
**Octa-peptides**			
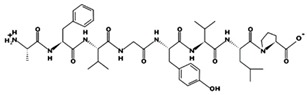	AFVGYVLP 18.02 μM [66] 864.4731 5.23 0 +5.10	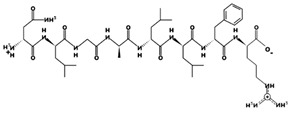	NLGALLFR - 902.5323 10.60 +1 +6.75
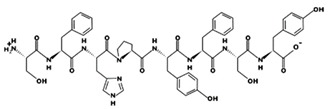	SFHPYFSY 82.71 μM [66] 1046.4484 7.57 0 +6.45	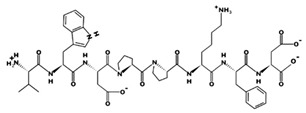	VWDPPKFD 9.1 μM [44] 1002.4796 3.91 −1 +14.00
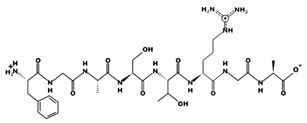	FGASTRGA 14.7 μM [68] 765.3759 10.90 +1 +12.01	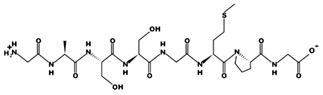	GASSGMPG 6.9 μM [73] 662.2685 5.60 0 +12.24
**Nona-peptides**			
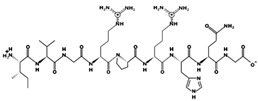	IVGRPRHQG 6.2 μM [60] 1018.5770 12.49 +2 +15.48	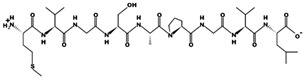	MVGSAPGVL 3.09 μM [67] 829.4354 5.51 0 +8.46
**Deca-peptides**			
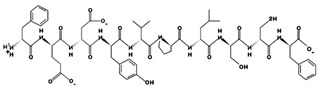	FEDYVPLSCF 11.26 μM [44] 1218.5249 2.92 −2 +9.91	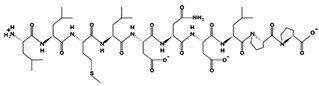	LLMLDNDLPP 35.7 μM [69] 1139.5877 2.76 −2 +10.64
			

**Table 8:** ACE inhibition activity of synthesized dried bonito thermolysin hydrolysate peptides and their fragments.

Synthesized Peptide	Fragment of the Synthesized Peptide	
IC_50_ (µM)		Activity Improved By
IVGRPRHQG	VGRPRHQG	
6.2	5.4	13%
IWHHTF	IW	
2.5	2.0	20%
IKPLNY	IKP	
43	1.7	96%
LKPNM	LKP	
17	1.6	90.5%
DYGLYP	LYP	
62	6.6	89%

Adapted from Yokoyama et al. [60].

**Table 9:** ACE inhibition and antihypertensive effects of dried bonito thermolysin hydrolysate peptides.

Sequence	Not pre-Incubated with ACE IC_50_ (µM)	Pre-Incubated with ACE IC_50_ (µM)	Decrease of SBP in SHR (60 mg kg^−^^1^ BW) (max, Δmm Hg)	Treatment Time (h)
**Inhibitor-type**				
IY	2.31	1.9	19	2
IKP	6.9	3.4	20	6
LKP	0.32	0.32	18	4
IWH	3.5	3.5	30	4
**Prodrug-type**				
LKPNM	2.4	0.76	23	6
IWHHT	5.8	3.5	26	6

Adapted from Fujita et al. [77].

**Table 10:** Drug-likeness and ADME (adsorption, distribution, metabolism, and excretion) analysis of selected anti-ACE and antihypertensive fish biopeptides by Lipinski’s rules.

	Lipinski’s rule of five					
	Molecular Weight (g mol^−1^)	Lipophilicity (MLog *P*)	Hydrogen Bond Donors	Hydrogen Bond Acceptors	No. of Rule Violations	Drug-Likeness
Sequence	Less than 500 Dalton	Less than 5	Less than 5	Less than 10	Less than two violations	Follows Lipinski’s rule
**Tri-peptides**						
AFL, FIA	~349	0.85	4	5	0	Yes
FQP	390.43	−0.55	5	6	0	Yes
IVW	416.51	0.68	5	5	0	Yes
LVL, VIL, IVL	~343	0.87	4	5	0	Yes
YLV, VIY	393.48	0.79	5	6	0	Yes
VAP	285.34	−0.53	4	5	0	Yes
VIF, IVF, FVL	~377	1.31	4	5	0	Yes
**Tetra-peptides**						
GPAV	342.39	−1.26	4	6	0	Yes
LAYA	436.5	−0.21	6	7	0	Yes
**Penta-peptides**						
ALPHA	507.58	−1.93	6	8	2	No
IWHHT	692.77	−2.76	10	10	3	No
LKPNM	601.76	−1.93	7	9	2	No
LYPPP	585.69	0.06	5	8	2	No
VELYP	619.71	−0.7	8	10	3	No
EHPVL	593.67	−1.85	7	10	3	No
EVLIQ	600.7	−1.34	8	10	3	No

**Table 11:** Antihypertensive fish peptides as active ingredients in commercial products.

Fish Name	Peptide Sequence	Product	Producer	Type of Product
Bonito (*Sarda orientalis*)	LKPNM	PeptACE™, Vasotensin®, Levenorm®, Peptide ACE 3000, Peptide Tea, Katsuobushi	Natural factors nutritional products Ltd., Canada, Metagenics, USA, Ocean nutrition Canada Ltd., Canada, Nippon supplement Inc., Japan	Tablet, capsule, powder, dietary supplement and functional food
Sardine (*Sardinops sagax*)	VY	Lapis Support, Valtyron®, Sato Marine Super P	Tokiwa Yakuhin Co., Japan, Senmi Ekisu Co., Japan, Sato pharmaceutical Co., Japan	Tablet, ingredient, beverage
Blue ling (*Molva dypterygia*)		Molval	Dielen, France	Nutritional supplement
Mackerel		Tensideal	ABYSS’ ingredients, France	Dietary supplement

Adapted from Gevaert et al. and Hayes et al. [98,99].
